# Hybrid micromagnetic and atomistic modeling of magnetization dynamics induced by engineered defects

**DOI:** 10.1038/s41598-025-31866-6

**Published:** 2025-12-21

**Authors:** Nastaran Salehi, Olle Eriksson, Johan Hellsvik, Manuel Pereiro

**Affiliations:** 1 Department of Physics and Astronomy, Uppsala University, Box 516, SE-751 20 Uppsala, Sweden; 2 WISE - Wallenberg Initiative Materials Science for Sustainability, Department of Physics and Astronomy, Uppsala University, SE-751 20 Uppsala, Sweden; 3 PDC Center for High Performance Computing, KTH Royal Institute of Technology, SE-100 44 Stockholm, Sweden

**Keywords:** Magnetic properties and materials, Spintronics, Physics, Condensed-matter physics, Topological matter, Topological defects

## Abstract

This study presents a 3D version of multiscale approach for investigating magnetization dynamics in multiscale, hybrid micromagnetic-atomistic simulations. The present work introduces engineered discontinuities (i) a double-slit structure, which enables the study of domain wall and spin wave interference, and (ii) a tetrahedron shaped cluster of atoms with tunable anisotropy, which provides insights into how localized anisotropic perturbations influence domain wall pinning and skyrmion stability in fully three-dimensional (3D) hybrid simulations. We considered the dynamics of spin waves, domain walls, as well as 3D skyrmions, in the presence of these defects. The magnonic double-slit experiment demonstrates interference patterns analogous to electronic wave phenomena, offering potential applications in wave-based computing. Additionally, the results reveal the impact of the local anisotropy that leads to distinct transformations, including domain wall deformations, tubular and spherical structures, skyrmion annihilation, and breathing mode. The findings underscore the critical role of defect-induced anisotropic interactions in controlling domain wall motion, skyrmion topology, and spin wave propagation.

## Introduction

The field of spintronics has gained significant interest due to its potential to improve information storage and processing technologies by using the spin degree of freedom of electrons^1–3^. In particular, domain walls and skyrmions have emerged as promising candidates for next-generation spintronic devices due to their unique topological properties, stability, and energy-efficient manipulation^4–8^, although skyrmion-based devices have not yet been commercialized. Domain walls and skyrmions provide robust mechanisms for data transport and logic operations^9–11^. However, the behavior of these magnetic structures in the presence of defects and localized anisotropy remains a crucial area of research, as material imperfections and engineered anisotropic regions significantly influence their dynamics^12^. To explore these effects, it is essential to move beyond ideal models and account for the role of defects and imperfections present in real materials.

While ideal magnetic systems offer promising device functionalities, real materials inevitably contain defects that can fundamentally alter their performance. Recent experimental studies have shown that introduced defects can be used to control magnetic textures. For example, atomic-scale vacancies and dislocations influence skyrmion stability^13^, artificial pinning centers guide domain wall motion^14^, and tailored anisotropy landscapes enable new switching mechanisms^15^. These studies reveal that defects are not merely imperfections but powerful tools for controlling magnetization dynamics. However, a systematic multiscale understanding of how atomic-scale defects influence mesoscale magnetic textures remains challenging, particularly for three-dimensional systems.

A key challenge in micromagnetic simulations is the need for multiscale modeling to accurately capture both atomic-scale interactions and large-scale magnetic texture evolution^16^. Multiscale approach is needed for structures or phenomena with atomic-scale length scales like narrow constrictions, tetrahedral anisotropy clusters, line or point defects, sharp domain-wall distortions, and atomistic-size skyrmions or vortices where local spin alignment, exchange, and defect-induced anisotropy must be resolved. In this work, we develop and apply the $\mu$-ASD module of the UppASD software^17^, a computational framework that integrates atomistic spin dynamics and micromagnetic simulations, enabling a comprehensive investigation of defect-induced effects. This investigation is focused on iron-iridium (Fe-Ir) thin films. The interatomic coupling interactions for these thin films are adopted from Ref^18^.. This approach allows us to explore localized engineered defect interactions while maintaining computational efficiency in larger-scale magnetic domains.

This study also represents an implementation of a fully three-dimensional (3D) multiscale simulation method, thus increasing its functionality beyond the previous two-dimensional (2D) implementation and analysis^19^. The present research extends the paradigm to explore 3D magnetization dynamics. This advancement facilitates a better comprehension of skyrmion behavior, domain wall interactions, and spin wave propagation within realistic, multilayered, and confined magnetic systems. As a result, and as demonstrated here, this progress opens up new prospects for multiscale modeling in 3D geometries.

We focus primarily on two type of engineered defects, namely a double-slit and an atomic cluster with tetrahedral geometry. A double-slit structure placed in the middle of the material serves as an ideal platform to investigate domain wall scattering and spin wave interference, similarly as in the case of the electronic double-slit experiment in optics and quantum mechanics. The double-slit experiment demonstrates the wave-particle duality of quantum particles, where a coherent wavefront passing through two slits produces an interference pattern on the other side. Similarly, in this work, we explore the magnonic counterpart of the double-slit experiment, where spin waves, excited by a microwave field, propagate through a magnetic medium containing a double-slit and generate an interference pattern characteristic of wave-like behavior. Additionally, we examine the dynamics of a domain wall crossing the double slit, where an external magnetic field is applied to drive the domain wall along the longitudinal direction. As the domain wall interacts with the spatial constraints of the slits, its trajectory and shape are altered. A dual analysis of spin wave interference and domain wall dynamics in a double-slit structure provides valuable insight into how structured engineered defects affect magnetization dynamics. The results contribute to the field of magnonics, where controlled interference of spin waves can be utilized for, e.g, information processing, magnonic transistors, and wave-based computing architectures^20,21^. This study not only establishes an intriguing link between quantum mechanical wave phenomena and magnonic wave dynamics but also provides a practical framework for designing spin wave-based logic and memory devices using engineered defect structures.

The second type of engineered defect considered in this study, as aforementioned, is an atomic cluster with tetrahedral geometry, with tunable anisotropy. Here, a localized tetrahedral anisotropic region is introduced to examine its effect on domain wall pinning and skyrmion dynamics. By systematically tuning the anisotropy strength and orientation (in-plane vs. out-of-plane orientation), we analyze how these modifications influence skyrmion and domain wall deformation^22^. Our results show that increasing the anisotropy in a small region (here described as a tetrahedron cluster) induces distinct magnetic texture responses, including domain wall elongation, skyrmion annihilation, and skyrmion breathing. Furthermore, the magnonic double-slit experiment reveals wave interference patterns that demonstrate the feasibility of controlling spin wave propagation using engineered defects. These findings contribute to a broader understanding of defect engineering in spintronic materials.

The structure of this paper is as follows. The results section presents a detailed analysis of the simulation outcomes, discussing the impact of localized anisotropic perturbations on domain walls, skyrmions, and spin waves. The discussion section provides concluding remarks and summarizing the implications of this study. In the methods section, we describe the method, detailing the Landau-Lifshitz-Gilbert (LLG)-based computational framework and the multiscale integration of atomistic and micromagnetic regions. Finally, the supplementary provides extended theoretical derivations, additional equations, and video visualizations depicting the time evolution of the studied magnetic textures^23^.

## Results

### A magnonic double-slit system

In this study, a double-slit described in the following is introduced in the middle of the system in the atomistic region, with the purpose of investigating the interference pattern of magnons.

#### Spin wave interference

The primary objective of this analysis is to examine the interference pattern of spin waves as they propagate through the slits, drawing an analogy to the classical electronic double-slit experiment in optics and quantum mechanics. In conventional electronic systems, the double-slit experiment demonstrates the wave-particle duality of electrons, revealing an interference pattern indicative of their wave-like behavior. Similarly, in this study, we aim to explore the magnonic equivalent of the double-slit experiment, where spin waves of collective spin excitations form interference pattern as they pass through the two slits.

**Fig. 1:**
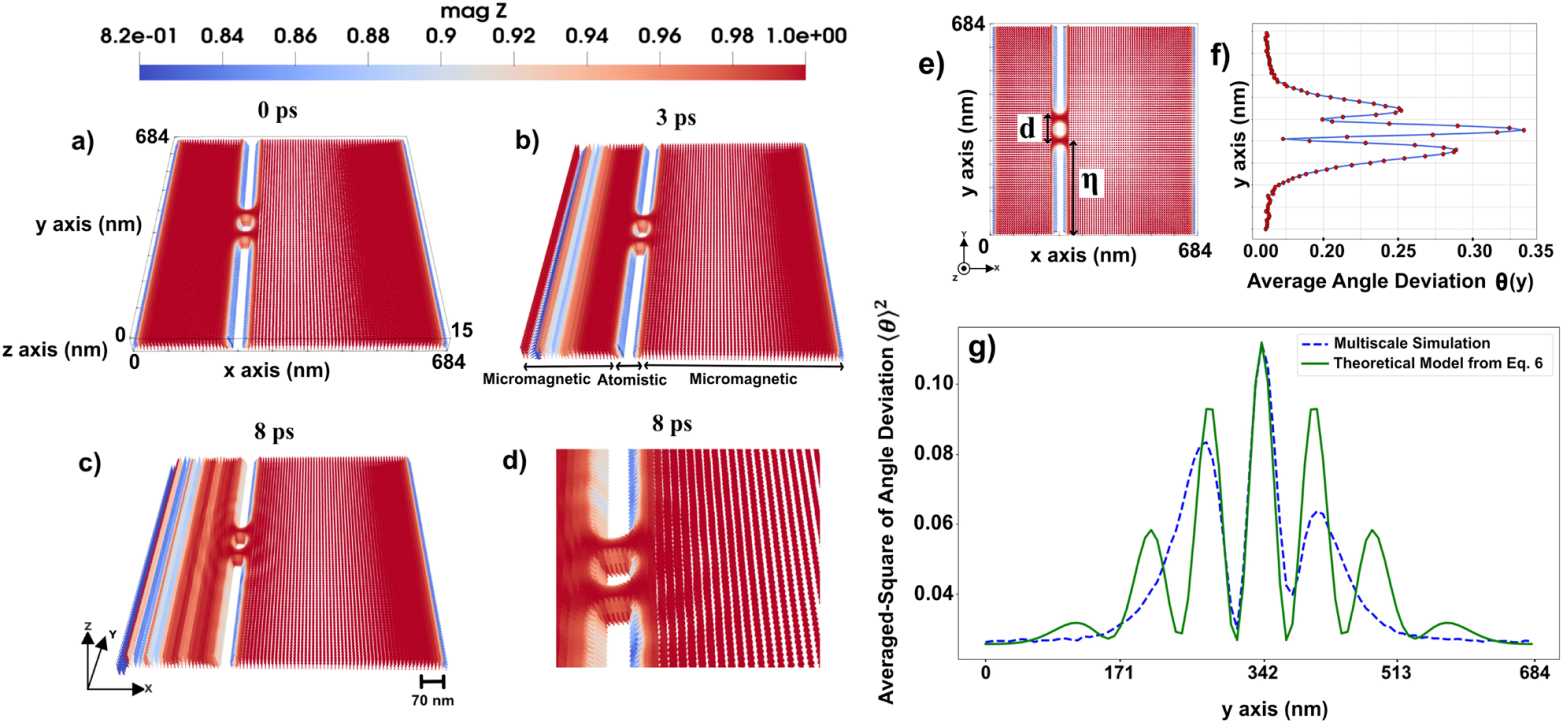
Spin wave propagation through a 3D double-slit. In (**a**) a 3D perspective of the system setup is illustrated together with the magnetic configuration in 0 ps time. In (**b** and **c**), we have 3 and 8 ps time frames of the system dynamics, respectively. A magnification of panel (**c**) around the slits is displayed in panel (**d**) to illustrate the interference pattern, (**e** and **f**) micromagnetic interference pattern induced by average angle deviation of the moment over time from *z*-direction, in degrees, as a function of the length along the *y*-axis (geometry shown in the **e**) at a chosen value of $x=311$ nm, $z=7.6$ nm, $d=76$ nm (the distance from the center to center of slits), and $\eta$=304 nm (the distance from $y=0$ nm to the center of the lower slit), (**g**) comparison of micromagnetic simulation data and the theoretical derived formula which shows interference pattern induced by averaged-square of angle deviation of the moment over time from *z*-direction, as a function of the length along the *y*-axis. The blue dashed line represent the micromagnetic data extracted from spin wave propagation, while the green line corresponds to the theoretical derived formula given by Eq. 6. The color code is showing the *z* component of normalized magnetization (red +1 and blue 0.82).

By analyzing the simulated interference patterns, this work provides fundamental insights into the wave nature of magnons and establishes parallels between quantum mechanical wave interference and magnonic wave dynamics, which develops new spin-based applications. The double-slit structure (shown in Fig. 1) serves as a controlled environment to study scattering effects and possible pinning mechanisms that arise due to spatially defined perturbations. In this part of the study, we investigate the propagation of spin waves through a double-slit configuration by applying a local oscillating microwave field to excite spin waves of the micromagnetic region. We include this term in LLG equation as^24^:

1 $$\begin{aligned} \textbf{H}_{{MW}}(t) = \textbf{H}_0 \cos (w t + \phi ) \end{aligned}$$

where $w=2\pi f$ is angular frequency and *f* stands for the microwave frequency. The time variable is defined by *t*. $H_0$ is the amplitude of the microwave field, and $\phi$ is the phase of the microwave field.

For the Fe-Ir system, the magnon excitations are in an energy range up to 100 meV^25^, which corresponds to a valid frequency range up to 24 THz. In our simulation, the excitation frequency is set to be 1 THz, ensuring a realistic representation of magnon dynamics within the system. The amplitude is set to be 1500 T, the pulse duration is defined as 300 ps, and the phase of the microwave field is chosen to be 1.5. The pulse is switched on and off at the proper time without using a Gaussian envelope. While the amplitude value used in simulations exceeds experimentally realistic magnitudes, it was intentionally set high to rapidly perturb spin configurations and thereby reduce the computational time. Additionally, this choice still preserves the underlying physics of the system. These parameters are optimized to effectively excite and sustain magnonic oscillations.

The field induced in Eq. 1 generates coherent spin waves, which travel through the micromagnetic system, interacting with the double-slit region in the middle of the system (see Fig. 1). The micromagnetic system under investigation has dimensions of $684 \times 684 \times 15~\text {nm}^3$. The atomistic region has the size of $38 \times 684 \times 15~\text {nm}^3$. The rectangular regions without magnetic moments, have the size of $19 \times 285 \times 15~\text {nm}^3$ (the upper and lower ones in Fig. 1) and $19 \times 38 \times 15~\text {nm}^3$ (the middle one in Fig. 1). The slits should be narrow enough to act as coherent sources and spaced optimally to produce a clear interference pattern, therefore the width of each slit is set to 38 nm. Slits are positioned at 216 nm in *x*-direction and the distance from center to center of the slits is 76 nm.

Figure 1 illustrates the spin wave propagation through the double slit. The dynamics of the system is presented in the Supplementary Video S1^23^.

The interference of spin waves traveling through the two slits is visible from Fig. 1 d) and seems to share common features to optical waves or the quantum mechanical double-slit experiment, but differs in the underlying physics and governing principles. In the quantum case, as specified by the Schrödinger equation, interference arises from the wavefunction of particles, whereas in spin waves, as results of the LLG equation, interference results from collective magnon excitations in a magnetically ordered medium that according to the results in Fig. 1 have wave-like properties.

**Fig. 2:**
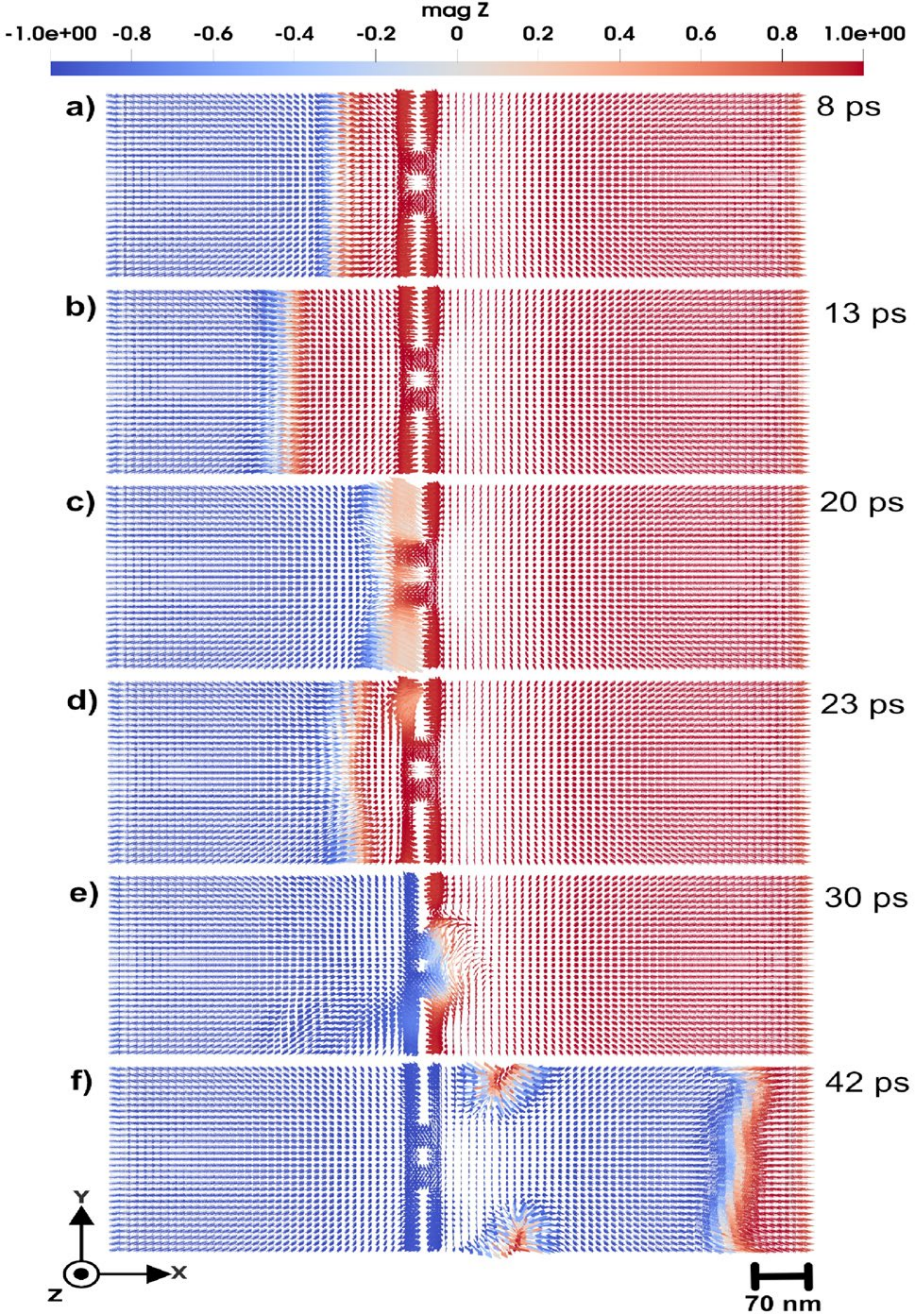
3D domain wall motion in the *x*-direction towards a double slit, induced by an applied external magnetic field. As the domain wall passes through the slits, it becomes perturbed, exhibiting a bouncing behavior for a period of approximately 25 ps. Panels (**a** to **f**) illustrate the evolution of the domain wall at different time frames, ranging from 8 to 42 ps. The color code shows the *z* component of the normalized magnetization.

Based on the micromagnetic simulation results of the double slit system, interference pattern is observed as illustrated in Fig. 1 f, which displays the interference pattern characterized by the average angular deviation of the magnetic moment over time from the *z*-direction, measured in degrees, as a function of the position along the *y*-axis. This analysis is conducted at a fixed coordinate of $x =311$ nm and $z = 7.6$ nm, where the intensity and peak of the interference pattern reach their maximum.

In order to make a proper comparison between the micromagnetic simulation results and theoretical predictions, we develop an analytical model for interference pattern of spin waves. In a ferromagnet with DM interaction, the energy of the spin wave can be written in the following expression^23^:

2 $$\begin{aligned} \hbar \omega ^c=D_{ex} q^2 - c D_{dm} q \end{aligned}$$

where *q* is the wave vector, $c=\pm 1$, $D_{ex}$ is the spin wave stiffness, and $D_{dm}$ is the DM stiffness. The wave vector of a given magnon frequency can be as:

3 $$\begin{aligned} q=\frac{c D_{dm}\pm \sqrt{D_{dm}^2\mp 4D_{ex}\hbar \omega }}{2D_{ex}} \end{aligned}$$

In this ferromagnetic medium, spin waves propagate where their phase coherence leads to interference patterns. We consider a spherical wave $W_{ext}$, generated by two point sources whose intensity decays exponentially as:

4 $$\begin{aligned} \textbf{W}_{\textrm{ext}}=W_0 \left( \frac{e^{i(q|\textbf{r}-\textbf{r}_1|-\omega t)}}{|\textbf{r}-\textbf{r}_1|} e^{|\textbf{r}-\textbf{r}_1|\xi }+\frac{e^{i(q|\textbf{r}-\textbf{r}_2|-\omega t)}}{|\textbf{r}-\textbf{r}_2|}e^{|\textbf{r}-\textbf{r}_2|\xi }\right) \end{aligned}$$

where $W_0$ is the amplitudes of the spherical waves, the coordinates of the vectors are given by: $\textbf{r}_1=(0,d+\eta )$, $\textbf{r}_2=(0,\eta )$, $\textbf{r}=(x,y)$, $\textbf{r}-\textbf{r}_1=(x, y-(d+\eta ))$ and $\textbf{r}-\textbf{r}_2=(x, y-\eta )$ (the setup and the reference frame is shown in Supplementary Fig. S1, S2 and S3^23^), and $\xi$ is the attenuation coefficient induced by the medium where the spin waves propagate, which can be derived as:

5 $$\begin{aligned} \xi ^c=\frac{\alpha \omega }{2 \frac{D_{ex}}{\hbar }q-c \frac{D_{dm}}{\hbar }} \end{aligned}$$

For small angle deviations, we can approximate the averaged-square of angle deviation by:

6 $$\begin{aligned} \langle \theta \rangle ^2\simeq \langle \frac{\gamma W_{ext}}{\omega } \rangle ^2 + \sigma =\left( \frac{\gamma }{\omega }\right) ^2\langle W_{ext}\rangle ^2 + \sigma \end{aligned}$$

where $\theta$ represents the average angle deviation of the moments from *z*-direction, $\gamma$ denotes gyromagnetic ratio, and $\sigma$ represents background spin intensity, potentially arising from microwave field-driven energy input. All the derivations for Eqs. 2-6 are provided in Supplementary, sections 1 and 2^23^. The parameters used in the spin wave model to predict the form of the diffraction pattern are collected in Supplementary Table S1^23^.

**Fig. 3:**
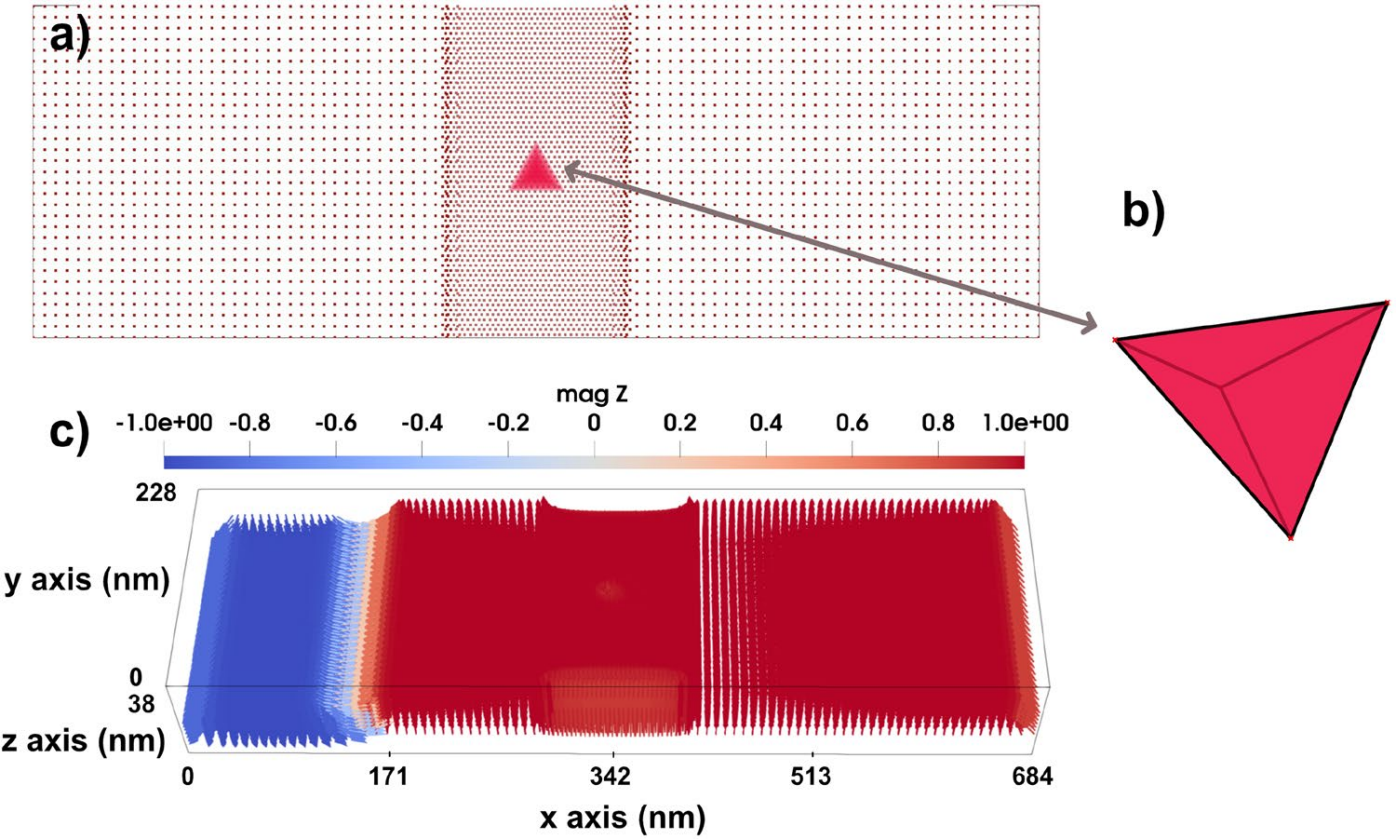
(**a**) Top-view of the structure of the system consisting of a rectangular atomistic region in the middle that contains tetrahedron cluster at its center. (**b**) A tilted view of the tetrahedron cluster. (**c**) A 3D structure of a system including a 3D domain wall, with an atomistic region in the middle which shows a higher spin density in the atomistic region compared to the surrounding micromagnetic part. The color bar is showing the *z* component of normalized magnetization.

This analogy suggests that spin wave mediated magnetization dynamics serve as a practical counterpart to the quantum mechanical double-slit experiment. As illustrated in Fig. 1 g, the fitted numerical results from Eq. 6, demonstrate that micromagnetic data follows the predicted theoretical form, indicating the wave nature of spin excitations in magnetic materials. The equation derived in this study effectively models the averaged-square of angle deviation of spin wave interference in a micromagnetic system. The resemblance to the quantum double-slit experiment is evident through the presence of interference fringes modulated by an exponential decay factor.

#### Domain wall scattering and acceleration

Double-slit configuration enables a focused analysis on how the domain wall structure and dynamics are influenced when passing through or interacting with geometrically confined defects of comparable scale. The 3D domain wall under investigation is a Néel-type wall with a width of approximately 11 nm ($\Delta = \sqrt{\frac{A_{e}}{K_u}}$) (see e.g. Ref^26^.). The size of the atomistic region is $38 \times 228 \times 38~\text {nm}^3$. Rectangular holes in this region have the size of $19 \times 76 \times 38~\text {nm}^3$ (upper and lower holes in Fig. 2) and $19 \times 23 \times 38~\text {nm}^3$ (the middle one in Fig. 2). The width of each slit is set to $\sim$ 26.6 nm. This configuration enables a focused analysis of how the domain wall structure and dynamics are influenced when passing through or interacting with geometrically confined defects of comparable scale. The double-slit structure also serves as a controlled environment to study scattering effects and possible pinning mechanisms that arise due to spatially defined perturbations.

**Fig. 4:**
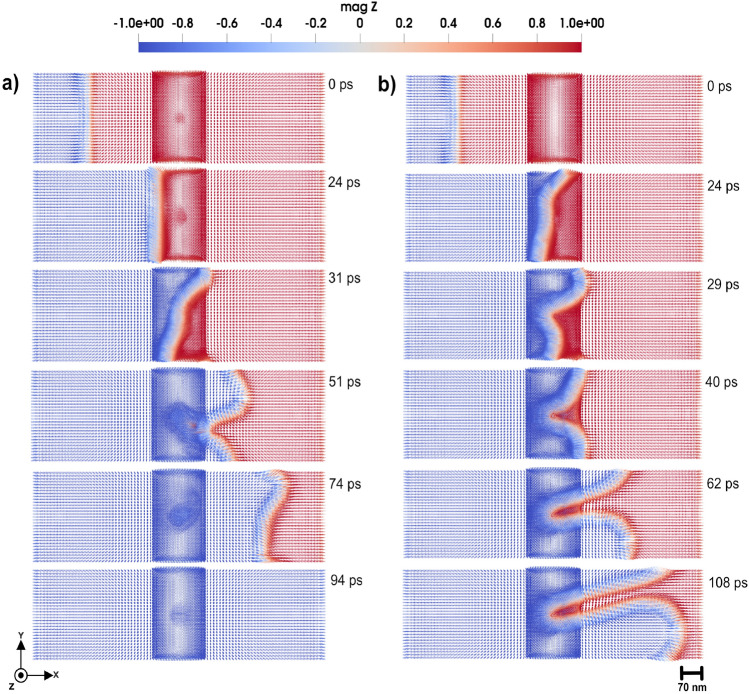
3D Domain wall motion by applying magnetic field of 0.47 T in negative *z*-direction. In the center of the atomistic region (shown by slightly deeper color) a local cluster is placed that has uniaxial anisotropy with (**a**) hard axis along the *z*-direction, with strength of 0.11 mRy, and (**b**) easy axis along *z*-direction and strength of 0.9 mRy. Simulations that consider a local anisotropy that has the *z*-axis as a hard axis in (**a**), produces similar results of panel (**a**). Also, by considering a local anisotropy in *z*-axis as a easy axis in (**b**), yields the same outcome of panel (**b**). The color bar is showing the *z* component of normalized magnetization.

As shown in Fig. 2, where snapshots of magnetic configurations are taken in a time period up to 42 ps, the application of an external magnetic field of 3.0 T in the *z*-direction induces the movement of the domain wall from the left to the right side of the system. Upon approaching the double-slit structure located in the middle of the system (see Fig. 2 a)-b)), the domain wall bounces. Despite this initial repulsion, the domain wall continues its forward motion, only to be repelled a second time as it interacts with the slit edges. Eventually, the domain wall successfully passes through the double-slit structure. However, while passing the slits (Fig. 2 c)-d)), the domain wall exhibits a pronounced curvature, arising from the spatial constraints imposed by the double-slit geometry and the localized magnetic interactions. This deformation underscores the influence of structural perturbations on domain wall dynamics, particularly in constrained environments. The domain wall motion under the applied magnetic field, offers a dynamic visualization of its behavior, including the moments of repulsion, re-approach, and eventual passing through the double slit. The dynamics of domain wall is presented in detail in Supplementary Video S2^23^.

In the presence of a double-slit defect, the dynamics of domain wall motion exhibit significant changes compared to motion in a defect-free system. Specifically, when the domain wall passes through the double slit, it experiences a significant acceleration. Quantitatively, the post-slit velocity of the domain wall increases to approximately twice the velocity observed during propagation in the absence of the double slit. This phenomenon suggests that the geometric confinement and localized magnetic interactions induced by the double slit act to compress and reconfigure the domain wall structure, resulting in reduced pinning and enhanced mobility beyond the slit.

We developed a one-dimensional (1D) model provided in Supplementary, section 3^23^, and Supplementary Fig. S4 and Fig. S5^23^, which explains the reason for the increase in velocity and explain the observed acceleration of magnetic domain walls as they traverse a double-slit geometry. To explain this behavior, we consider a 1D collective coordinate model describing the DW in terms of its center position *q*(*t*), width $\Delta (t)$, and magnetization angle $\phi (t)$. The geometric constriction is modeled as a localized potential centered at the slit position $q^\prime$, described by:

7 $$\begin{aligned} V(q-q\prime )=V_0 \; e^{-\frac{q-q\prime }{a}} \end{aligned}$$

where *a* is the width of the potential and $V_{0}$ is the maximum value of potential.

This potential stores elastic energy due to domain wall compression. When deriving the equations of motion via the Lagrangian formalism, including dissipation effects, the resulting velocity expression for the domain wall is:

8 $$\begin{aligned} \dot{q}&= \frac{1}{1+\alpha ^2} \left( \alpha \gamma \Delta H - \alpha \Delta \eta e^{-\left( \frac{q-q'}{a}\right) ^2} (q-q') - \alpha ^2 \dot{\Delta } \ln \sqrt{2} \right. \nonumber \\&\quad \left. + \frac{\pi D \gamma }{4\Delta \mu _0 M_s} \cos \phi + \gamma \Delta H_k \sin (2\phi ) \right) \end{aligned}$$

where $\mu _0$ is the vacuum magnetic permeability, $M_s$ is the saturation magnetization, *D* is the DM interaction, $K_0$ is the uniaxial anisotropy, *K* is the second-order uniaxial transverse anisotropy, *H* is an external magnetic field, $H_k=\frac{K}{\mu _0 M_s}$ is the uniaxial anisotropy field and $\eta =\frac{\gamma V_0}{\mu _0 M_s a^2}$.

Equation 8 reveals the physical origins of acceleration. The chirality introduced by the DM interaction influences DW dynamics, with the direction and magnitude of its effect governed by the sign of the DM interaction constant *D*. A positive *D* enhances DW velocity, while a negative *D* tends to suppress it. Nonetheless, in the context of the double-slit geometry, the dominant mechanism responsible for the observed velocity increase stems from the interaction with the slit potential. Specifically, the second and third terms in Eq. 8 play a central role.

The second term in Eq. 8, arising from the gradient of the localized potential, provides a forward driving force as the DW approaches the slit ($q - q\prime < 0$), resulting in an initial acceleration. Although this is followed by a decelerating effect once the wall passes the slit center ($q - q\prime> 0$), the structural modification imparted during the approach is critical. This interaction leads to a sudden compression of the DW, manifesting as a rapid decrease in the domain wall width ($\dot{\Delta } < 0$). As a result, the third term in Eq. 8 becomes positive and contributes an additional boost to the DW velocity after it exits the slit. This two-stage mechanism that consists of initial acceleration via geometric potential and post-slit acceleration through width contraction is responsible for the observed doubling of the velocity of the domain wall beyond the slit. Consequently, the double-slit structure effectively serves as a dynamic enhancer of domain wall speed, demonstrating a mechanism for controlling and tuning domain wall velocity through engineered nano-structures.

As a final comment to this section, we note that from an experimental perspective, the detection of domain wall dynamics (Fig. 2) is more feasible than observing the small dynamical components of spin waves (Fig. 1). Magneto-optical Kerr effect (MOKE) and related techniques provide strong contrast for domain wall motion, since the magnetization varies from $+1$ to $-1$ across the wall, making it relatively straightforward to measure. In contrast, detecting magnonic interference patterns remains more challenging, as the variations are much weaker. Nevertheless, the double-slit geometry discussed in Fig. 1 remains conceptually interesting as an analogue of optical and quantum interference, and provides a theoretical framework for exploring how engineered defects can control spin-wave propagation.

### Effects due to a tetrahedral anisotropy cluster

Variation in magnetocrystalline anisotropy within a magnetic material can occur as a result of heterogeneity in its composition or can be artificially engineered through various processing methods. It is experimentally accessible to embed small crystalline regions (inclusions) within a magnetic host system, where these inclusions exhibit different magnetocrystalline anisotropy owing to distinct crystal orientations, phases, or compositions. Such heterogeneity leads to spatial variations in magnetic anisotropy magnitudes relative to the surrounding material. Experimentally, stress annealing is a widely used technique in Fe-based nanocrystalline alloys, where applying tensile stress during annealing induces magnetic anisotropy aligned with the stress direction by altering magnetoelastic interactions^27^. Moreover, hydrogenation of metallic multilayers such as Co/Pd interfaces can locally modulate magnetic anisotropy by altering the interfacial electronic structure, leading to spatially confined variations in anisotropy energy^28^. To experimentally detect and map local magnetic anisotropy, techniques such as magnetic force microscopy (MFM), scanning Hall probe microscopy, and polarized neutron diffraction are employed^29^. These allow for spatially resolved imaging of magnetic domains and anisotropy tensors, thereby revealing localized variations in magnetic behavior within heterogeneous or treated systems. Collectively, both material design and experimental treatments offer routes to achieve and characterize local magnetic anisotropy in metallic systems, enabling the development of advanced magnetic devices with region-specific magneto-crystalline anisotropies.

**Fig. 5:**
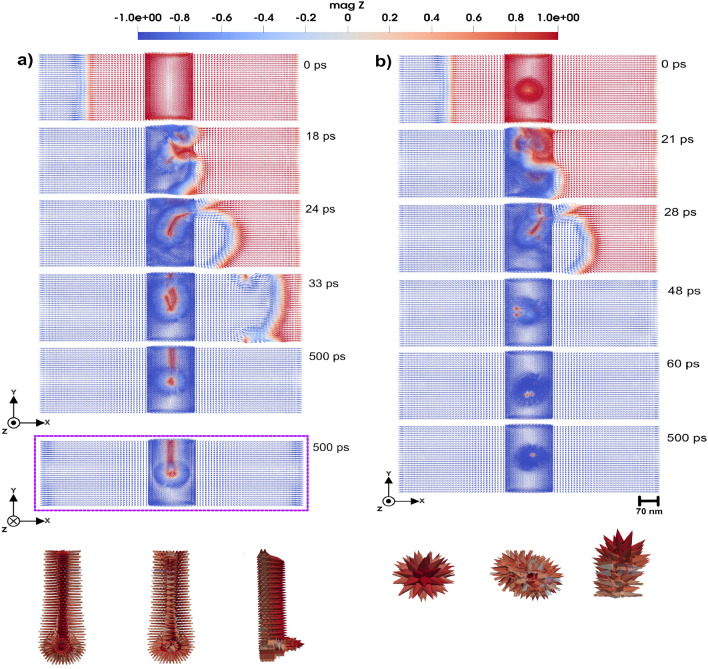
3D Domain wall motion by applying a magnetic field of 1.5 T through a system with a tetrahedron cluster with uniaxial anisotropy that has (**a**) easy axis along *z*-direction, (**b**) hard axis along *z*-direction and strength of 0.9 mRy which panels represent a top view of the magnetic texture along the *z*-direction, and a bottom view along the same direction for (**a**), elucidating the shape of the created skyrmion in three dimensions. Bottom panels in (**a**) illustrate the bottom, top, and lateral views of the 3D skyrmion, each exhibiting a 90-degree bend, while in (**b**) illustrate a generated hedgehog skyrmion from the top, bottom, and side views, respectively. The color bar is showing the *z* component of normalized magnetization.

Local lattice imperfections are also considered in this set of simulations. In particular, we consider a defect region with a tetrahedral geometry, as illustrated in Fig. 3. The micromagnetic system under investigation has dimensions of $684 \times 228 \times 38~\text {nm}^3$. The atomistic region has the size of $114 \times 228 \times 38~\text {nm}^3$. The tetrahedral cluster in the middle of the atomistic region contains approximately 720 atoms arranged in nine layers. Its overall dimensions are $38 \times 32.6 \times 30.7~\text {nm}^3$. This cluster is assigned an anisotropy value different from the rest of the system, whereas all other simulation parameters are kept the same. Along the *y*-direction, the system has a periodic boundary condition. By systematically tuning the anisotropy of this local cluster, we illustrate its potential in influencing the dynamics of the domain wall and skyrmion. We have to mention here that, in an experimental realization, the corners of the tetrahedral inclusion would not be atomically sharp but slightly rounded due to surface relaxation and fabrication limits. However, we do not expect this to qualitatively affect our conclusions. The purpose of using an ideal tetrahedral geometry in the simulations is to provide a well-defined and symmetric model system that allows us to isolate the influence of local anisotropy strength and orientation.

#### Domain wall pinning and transformation

The 3D domain wall under investigation here has a width of $\sim$ 11.4 nm. By application of an external magnetic field in the negative *z*-direction, the domain wall starts to move from the left to the right side of the simulation box. In this study, we systematically tuned the uniaxial anisotropy of a smaller volume in the atomic region, to investigate its impact on the domain wall dynamics under two distinct conditions: i) with the local anisotropy aligned along the easy axis of the micromagnetic region (the *z*-axis of the simulation box) and ii) with local anisotropy aligned along the hard axes of the micromagnetic region (the *xy*-plane of the simulation box). The uniaxial anisotropy values explored for the local impurity were 0.11 mRy, 0.9 mRy, and 1.5 mRy. These values were chosen so that the first value (0.11 mRy) was very close to the anisotropy constant of the system (0.058 mRy) while the other ones were progressively increased in order to emphasize the influence of the tetrahedron anisotropy as compared with the host material. The outcomes of the simulations reveal a complex interplay between the anisotropy strength, its directional alignment, and the domain wall behavior as it interacts with the tetrahedral region. This approach offers a pathway to investigate how local anisotropy variations can alter domain wall properties such as width, pinning potential, and mobility.

**Fig. 6:**
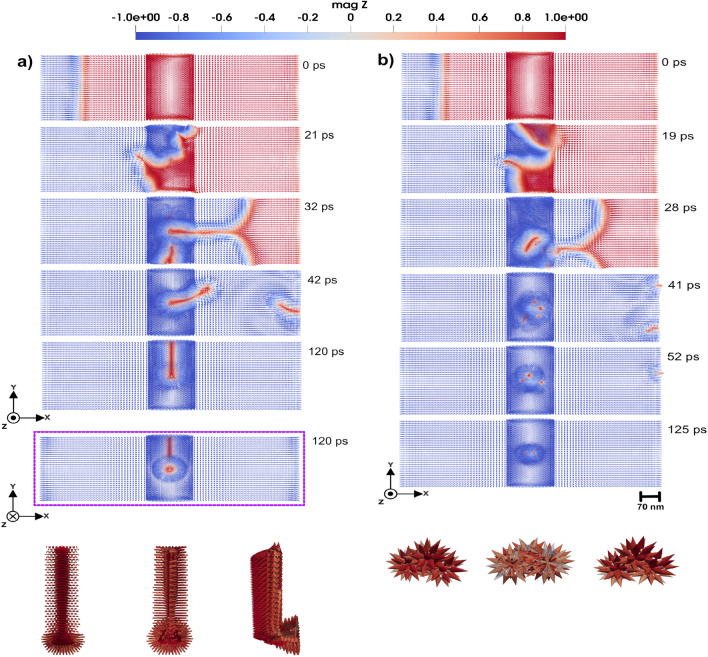
3D Domain wall motion by applying a magnetic field of 2 T so that a domain wall moves through the tetrahedron cluster with uniaxial anisotropy that has (**a**) easy axis along *z*-direction, (**b**) hard axis along *z*-direction and strength of 1.5 mRy. Panels represent a top view of the magnetic texture along the *z*-direction, and a bottom view along the same direction for a, elucidating the shape of the created skyrmion in three dimensions. Bottom panels in (**a**) respectively illustrate the bottom, top, and lateral views of the 3D skyrmion, each exhibiting a 90-degree bend while in (**b**) illustrate three generated hedgehog skyrmions from the top, bottom, and side views, respectively. The color bar is showing the *z* component of normalized magnetization.

We first considered a uniaxial anisotropy of the defect with the tetrahedral region set to 0.11 mRy. We investigate two directions for the anisotropy: along the easy (*z*-direction) and hard axis (*x*-*y* plane). In Fig. 4 a, the tetrahedron has anisotropy along hard axis (*x*-*y* plane). By applying a magnetic field of 0.47 T in negative *z*-direction, the domain wall successfully passes from left to right in the simulation cell, as shown in Fig. 4 a. However, due to the influence of the tetrahedron topology and magnetic properties, the domain wall experiences slight disturbances, manifesting as localized deformations and pinning during the time evolution. This phenomenon occurs when the local anisotropy is aligned along the easy axis of the micromagnetic region, as well as when the local anisotropy is along the hard axis of the micromagnetic region, as illustrated in Supplementary Video S3 and S4^23^.

**Fig. 7:**
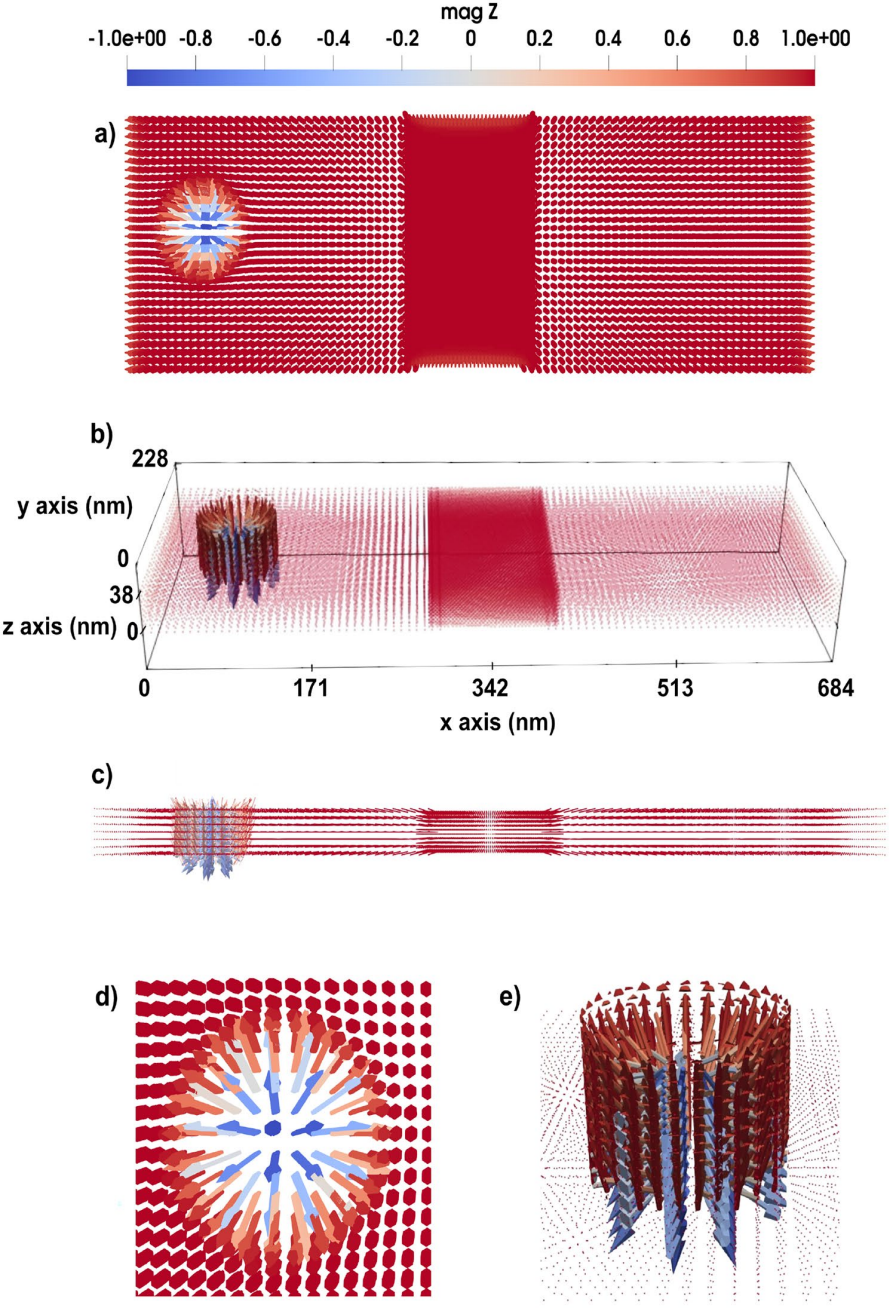
The magnetic texture of a 3D skyrmion. From panels (**a** to **e**), various perspectives of the 3D skyrmion are presented, highlighting its tubular magnetic configuration, which resembles a Neél-type structure observed in 2D magnetic systems. Panel (**a**) represents a top view, whereas panels (**b** and **c**) provide a lateral view. Panels (**d** and **e**) show the skyrmion magnified, providing detailed information about the magnetic texture. The color bar is showing the *z* component of normalized magnetization.

These findings can be directly linked to the microscopic origin of the Barkhausen effect, as previously discussed in Ref^19^.. In the present study, the tetrahedral impurity region serves as a localized anisotropy-induced defect, which behaves similarly to irregularities in the lattice such as dislocations or impurities. As the domain wall interacts with this region, it experiences localized pinning, where components of the wall become temporarily stuck due to the strong energy barrier associated with anisotropy. Similar to the behavior illustrated in earlier studies where parts of the domain wall are trapped by defects, the pinned region moves slowly behind the rest of the wall. The following unpinning phenomena, especially under increased magnetic field strength, are similar to the sudden jumps in magnetization observed experimentally in the Barkhausen effect, highlighting how atomic-scale anisotropy variations can lead to abrupt, collective magnetic changes. The atomistic modeling approach adopted here provides a deeper insight into such phenomena, capturing the complex interplay of local magnetic parameters at the atomic level.

As the uniaxial anisotropy of the impurity region is increased to 0.9 mRy, the interaction with the domain wall becomes more pronounced, as one can see in Fig. 4 b. By applying an external field of 0.47 T in negative *z*-direction, the domain wall moves from left to right in the simulation cell. Upon approaching the impurity region, the domain wall becomes pinned and takes a significantly longer time to pass the region. In fact, as Fig. 4 b shows, during the time of the simulations the domain wall has a part that never leaves the impurity region. This behavior occurs for the both cases when anisotropy is along easy and hard axis of the micromagnetic region as shown in Supplementary Video S5 and S6^23^. This is attributed to the stronger anisotropy-induced energy barrier, which opposes to the domain wall motion.

**Fig. 8:**
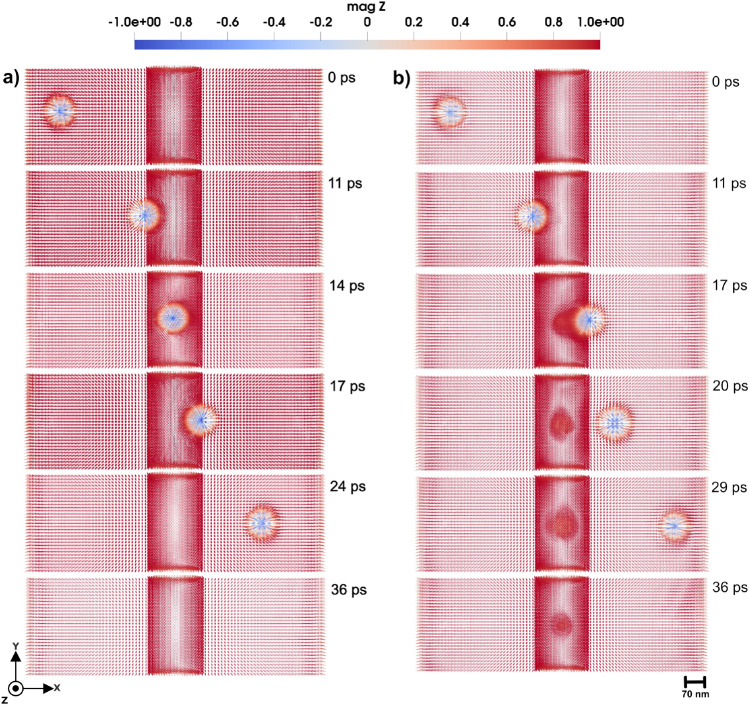
3D Skyrmion motion by applying a STT of 30 m/s through the simulation cell, with a defect region provided by a tetrahedron cluster with uniaxial anisotropy. (**a**) The easy axis is along the *z*-direction and (**b**) the hard axis is along the *z*-direction and the uniaxial anisotropy constant is 0.11 mRy. The color bar is showing the *z* component of normalized magnetization.

**Fig. 9:**
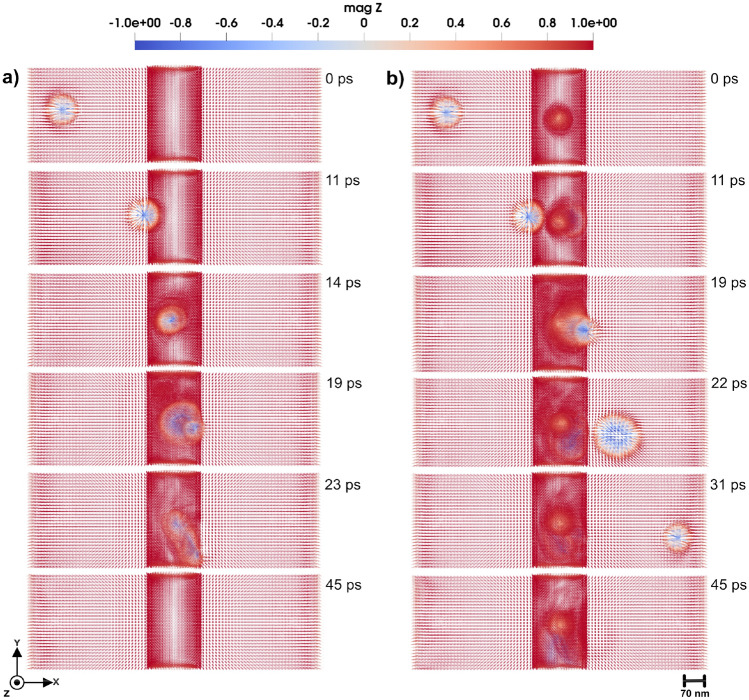
3D Skyrmion motion by applying STT of 30 m/s through the simulation cell, with a defect region shaped as a tetrahedron cluster with uniaxial anisotropy. (**a**) The easy axis is along the *z*-direction and (**b**) the hard axis is along the *z*-direction with the strength of 0.9 mRy. The color bar is showing the *z* component of normalized magnetization.

When the applied field is increased to 1.5 T, the domain wall can overcome the impurity region after being pinned to it but the domain wall exhibits dramatically different behaviors depending on whether the anisotropy is aligned along the easy or hard axis of the micromagnetic region. In the case where the impurity uniaxial anisotropy is along the easy axis of the micromagnetic region, an external magnetic field of 1.5 T in negative *z*-direction causes the domain wall to approach the impurity region, where begins to lose its coherent structure (see Fig. 5 a). This leads to the emergence of stable tubular configurations, as shown in Fig. 5 a. This tubular structure persists and stabilizes, suggesting that the strong easy-axis anisotropy introduces a localized energy landscape that fundamentally alters the domain wall configuration. The dynamics of this domain wall is presented in Supplementary Video S7^23^. Interestingly, the domain wall dynamcis of Fig. 5 a serves as an initial step for skyrmion formation, and in this scenario, the generated 3D skyrmion exhibits a 90-degree bending. This is illustrated in bottom panels in Fig. 5 a, where different projections of the skyrmion structure are illustrated.

**Fig. 10:**
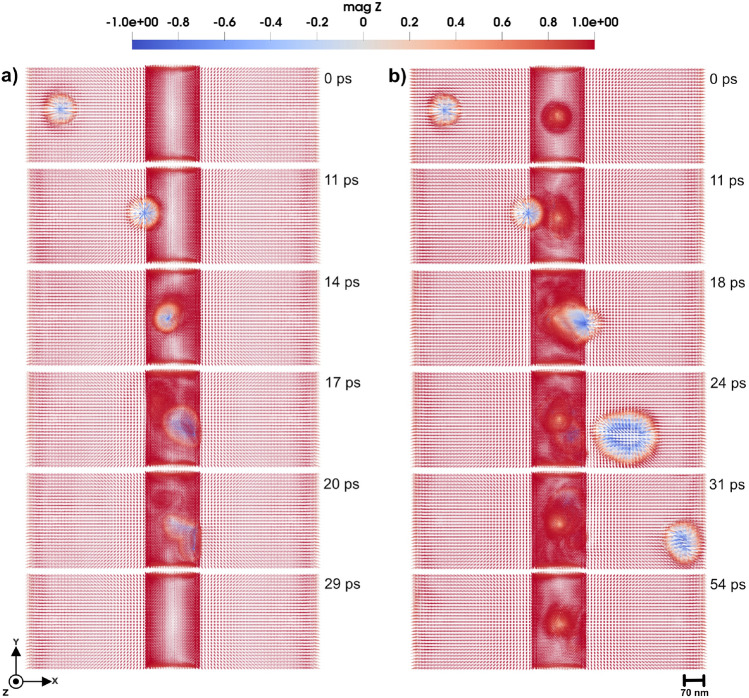
3D Skyrmion motion by applying STT of 30 m/s through the simulation box, with a defect region shaped as a tetrahedron cluster with uniaxial anisotropy. (**a**) The easy axis is along the *z*-direction and (**b**) the hard axis is along lying in *x*-*y* plane with strength of 1.5 mRy. The color bar is showing the *z* component of normalized magnetization.

The 90-degree bent skyrmion has potential applications in magnon transport. Given this unique curvature, it is plausible that magnons could be excited at the bottom of the system and subsequently guided along the bent domain wall, effectively channeling spin waves through a non-linear path. Such a configuration could serve as a waveguide for magnons, enabling controlled spin wave propagation and bending, that could become a crucial aspect for future magnonic circuits and information carrying technologies^20,21,30,31^.

By having uniaxial anisotropy of the defect region aligned along the hard axis of the micromagnetic region with a strength of 0.9 mRy, and applying field of 1.5 T, the domain wall also loses its coherence upon entering the tetrahedral region, as presented in Fig. 5 b and Supplementary Video S8^23^.The disturbance results in the formation of a tubular hedgehog skyrmion configuration shown in Fig. 5 b. This structure remains stable after its formation, highlighting a distinct anisotropy-dependent response.

The triangular tetrahedral cluster influences the local magnetization dynamics by inducing domain wall distortions and skyrmion deformations. In particular, we observe the formation of bent and tubular spin textures, as well as variations in the skyrmion number depending on the anisotropy strength and orientation. Such an effect could enable localized magnon control, which is essential for magnonic computing and low-power spintronic applications^20,32,33^. These results highlight how localized anisotropy variations can directly control the topology and stability of magnetic textures in three-dimensional systems^19^.

With the anisotropy further increased to a high value of 1.5 mRy, the domain wall dynamics remain qualitatively similar to those observed at 0.9 mRy, by applying a 2 T external magnetic field in the negative *z*-direction. When the uniaxial anisotropy within the tetrahedral region is aligned with the easy axis of the surrounding micromagnetic region, applying field drives the domain wall toward the impurity and begins to lose its coherent structure (see Fig. 6 a). The tubular structure illustrated in Fig. 6 a, remains stable over time. This configuration indicates that in the same case as previous shown data, the strong easy-axis anisotropy creates a localized energy landscape that significantly modifies the domain wall structure. The detailed evolution of this domain wall can be seen in Supplementary Video S9^23^.

When the uniaxial anisotropy within the defect region is oriented along the hard axis of the surrounding micromagnetic region, the domain wall again loses its coherence upon entering the tetrahedral region, as shown in Fig. 6 b and Supplementary Video S10^23^. This deformation gives rise to three distinct hedgehog structures(see Fig. 6 b).

When the domain wall passes through the defect, we observe clear changes in the skyrmion number depending on both the strength and direction of the magneto-crystalline anisotropy of tetrahedron. Considering the anisotropy of the tetrahedron to be equal to 0.9 mRy, when anisotropy is along the easy axis of the micromagnetic region, the skyrmion number reaches a value of $\bar{Q}^V=$ –40. But when the anisotropy is along the hard axis, the value drops down to $\bar{Q}^V=$ –26. Increasing the anisotropy of the tetrahedron to 1.5 mRy in the easy axis direction, it leads a bit to an increase in the skyrmion number, reaching $\bar{Q}^V=$ –46 with respect to the case with $K_a=0.9$ mRy. Interestingly, when the anisotropy constant is 1.5 mRy along the hard axis, the value stays $\bar{Q}^V=$ –26, showing no change. The higher order skyrmions generated in the current setup provides a clear indication about the complex magnetic texture shown in Figs. 5 a)-6 b).

These results suggest that easy-axis anisotropy in the inclusion tends to favor more twisting in the spin texture as the domain wall interacts with the defect, likely because it supports out-of-plane alignment of spins. That allows for more complex, topologically rich structures to form, around the distorted region introduced by the tetrahedron. On the other hand, hard-axis anisotropy seems to suppress that kind of twisting, so the topological charge stays lower and does not vary much even when the anisotropy strength is increased. It appears that once anisotropy reaches a level where spin canting is strongly suppressed, further enhancement has negligible influence. These observations underscore the significant role of localized anisotropy variations in dictating the behavior of magnetic textures such as domain walls and formation of topological structure. Using a single micromagnetic node to model the tetrahedral defect erases key multiscale effects and yields a straight, undeformed domain wall. Resolving atomistic-scale anisotropy gradients is crucial, so a hybrid model with an explicit atomistic defect region is necessary to capture the correct physics.

#### Skyrmion-defect interactions

A 3D skyrmion is generated in the micromagnetic region, by applying a local magnetic field in the negative *z*-direction as an external excitation. We used a 300 T local field to tilt the direction of the local magnetic moments in the opposite direction of the neighboring atoms with the aim to generate the 3D skyrmion quickly and prevent a time consuming simulation. This field modulates the local magnetization, leading to the formation of a tubular skyrmion in 3D, a topologically stable structure characterized by a swirling magnetization pattern along the depth of the system as is illustrated in Fig. 7.

Once the skyrmion is generated and stabilized, it can be moved by applying a spin transfer torque (STT), a phenomenon where the angular momentum of a spin-polarized current influences the local magnetization dynamics. The STT effect is included in the LLG equation of the micromagnetic region, via an additional adiabatic term^34,35^:

9 $$\begin{aligned} \textbf{H}_{STT} = -(\textbf{u} \cdot \nabla )\textbf{m} \end{aligned}$$

where $\textbf{H}_{STT}$ is the STT effective field, the vector *u* is expressed in velocity units and is directly proportional to the applied current velocity:

10 $$\begin{aligned} \textbf{u} = \frac{Pg\mu _{B}}{2eM_s}\textbf{j}_{e} \end{aligned}$$

where *P* is polarization, *g* is the Lande factor, $j_e$ is electron current density, *e* is the electron charge and $M_s$ is saturation magnetization.

In this section, we explore the behavior of a 3D skyrmion as it interacts with a tetrahedral region featuring a change in the anisotropy values. The skyrmion motion is driven by STT, that is induced by a spin current applied from the left of the simulation cell, causing the skyrmion to move towards the right. The velocity of the skyrmion due to the STT depends on the parameters of Eq. 10, and is tuned to ensure the controlled and observable motion. As the skyrmion approaches to the tetrahedral region, its interaction dynamics are analyzed under different anisotropy strengths and orientations (easy or hard axis). The skyrmion response is significantly different, depending on the easy axis orientation of the local defect region. With the tetrahedral anisotropy set to 0.11 mRy and aligned along the easy axis, the skyrmion, driven by a STT with velocity 30 *m*/*s*, traverses the region without notable deformation or disruption as shown in Fig. 8 a), and Supplementary Video S11^23^. When the anisotropy is instead aligned along the hard axis, the skyrmion still passes through the tetrahedral region(see Fig. 8 b)), however, exhibiting a temporary and small size increase (diameter increases $\approx$ 23 nm) during and shortly after the interaction. The dynamics of this skyrmion is presented in Supplementary Video S12^23^.

Unlike domain walls, which exhibit curvature and pinning, due to the interaction provided by the tetrahedral region, the skyrmion maintains its structural integrity. This resilience is attributed to the inherent stability provided by the skyrmion topology, which enables it to navigate low-anisotropy regions with minimal perturbation. At a higher anisotropy of 0.9 mRy and with a STT applied along the *x*-direction with a velocity of 30 *m*/*s*, the skyrmion motion through the tetrahedral region slows down. When the anisotropy of the cluster is aligned along the easy axis of micromagnetic region, the moving skyrmion disappears when it reaches the tetrahedral region, as illustrated in Fig. 9 a) and Supplementary Video S13^23^. This disappearance suggests that the strong easy-axis anisotropy creates a local energy barrier landscape that destabilizes the skyrmion core, effectively destroying its topological structure.

With the anisotropy of the tetrahedron aligned along the hard axis, the skyrmion again successfully passes the tetrahedral region, however, it has an increase in its size as shown in Fig. 9 b). The dynamics of this skyrmion is presented in Supplementary Video S14^23^.

At the highest defect anisotropy of 1.5 mRy, by applying STT with velocity 30 *m*/*s*, the skyrmion exhibits similar behavior to that observed at 0.9 mRy. When the anisotropy of the cluster is aligned along the easy-axis, the skyrmion disappears upon entering the tetrahedral region, as shown in Fig. 10 a) and Supplementary Video S15^23^.

With the cluster anisotropy aligned with the hard axis, the skyrmion traverses the tetrahedral region, exhibiting a temporary expanding in size during the interaction, as illustrated in Fig. 10 b). This temporary size modulation is consistent with a breathing mode, a dynamic mode where the skyrmion radius oscillates in time due to perturbations in the local magnetic energy landscape^36–39^. In this case, the abrupt change in anisotropy energy within the tetrahedron acts as a perturbative factor, inducing a nonlinear response of the skyrmion internal structure, leading to temporary expansion before relaxing back to its equilibrium size as shown in Fig. 10 b). The skyrmion gradually returns to its original size, indicating a recovery of its initial configuration once it exits the high-anisotropy area. The dynamics of the system is presented in Supplementary Video S16^23^. This behavior suggests that engineered anisotropy regions can serve as control points for skyrmion deformation and energy storage, which are crucial for applications in skyrmion-based memory and logic devices where controlled skyrmion dynamics are essential.

By decreasing the velocity of STT, the same trend remains as presented in Supplementary Fig. S6 and S7^23^ and Supplementary Video S17, S18, S19 and S20^23^.

Therefore, in the cases where the anisotropy of the cluster is aligned along the hard axis of the micromagnetic region, and for varying anisotropy strengths of 0.11, 0.9, and 1.5 mRy, the skyrmion exhibits an increase in size after traversing the cluster and undergoes a breathing mode. In contrast, when the cluster anisotropy is oriented along the easy axis of the micromagnetic region, the skyrmion passes through the defect without any noticeable change in size or shape for low anisotropy values. However, at higher anisotropy strengths, the skyrmion is annihilated upon encountering the defect.

## Discussion

In this work, we have developed and applied a fully 3D multiscale modeling approach to investigate the influence of defects on magnetization dynamics in hybrid micromagnetic-atomistic systems. By employing the $\mu$-ASD module, we explored the interplay between domain walls, skyrmions, and engineered local anisotropies in Fe-Ir thin film systems. Our simulations demonstrate that localized magnetic inhomogeneities, modeled as geometrically confined anisotropic clusters, exert a profound impact on both static and dynamic magnetic configurations.

The double-slit structure enabled the observation of spin wave interference fringes with wavelength modulations on the order of 76 nm, in quantitative agreement with analytical predictions based on wave theory (Fig. 1) with a coefficient of determination $R^2 \approx 0.7$. This finding is analogous to quantum mechanical systems, reinforcing the wave nature of magnons in confined geometries. The results of this study contribute to the broader field of magnonics, where spin waves are used for information processing and wave-based computing. Understanding spin wave interference in structured media is crucial for developing next-generation magnonic logic devices, wave-based transistors, and nonvolatile spin wave memory, where information is encoded in the phase and amplitude of propagating magnons rather than in electron charge. Additionally, domain wall dynamics in the presence of such slits revealed transient pinning and curvature effects, including enhanced post-slit acceleration explained through an effective energy-based model (Fig. 2).

In the case of tetrahedral defect clusters with tunable anisotropy, we observed complex domain wall responses ranging from minor deformations and Barkhausen-like pinning (Fig. 4) at low anisotropy ($K_u \approx 0.11$ mRy) to complete topological transformations at higher values ($K_u \ge 0.9$ mRy). Notably, the formation of $90^\circ$-bent (Fig. 5 a), 6 a)) tubular skyrmion-like textures (Fig. 5 b), 6 b)) was observed, illustrating how local anisotropy gradients can be utilized to create magnetic textures with non-trivial shape. Furthermore, 3D skyrmion dynamics under spin-transfer torque exhibited sensitivity to defect-induced anisotropy. Soft easy-axis anisotropic regions ($K_u^{easy} \approx 0.11$ mRy) permitted smooth skyrmion motion (Fig. 8 a)), while strong easy-axis anisotropy ($K_u^{easy} \ge 0.9$ mRy) led to topological breakdown which lead to skyrmion disappearing (Fig. 9 a), 10 a)), and hard-axis anisotropy caused temporary size modulation consistent with skyrmion breathing modes (9 b), and 10 b)).

This study reinforces the concept of topological protection in magnetic skyrmions. Skyrmions displayed minimal deformation as they traversed impurity regions in our simulations. In contrast, domain walls, which lack a non-trivial topological character, are severely perturbed by defect structures, often becoming pinned, distorted, or even deformed. These observations align with the theoretical understanding that the non-zero topological charge of skyrmions imparts a degree of robustness against local perturbations, which is essential for their reliable function in spintronic applications where disorder and imperfections are inevitable.

These findings also demonstrate that localized anisotropic perturbations can reconfigure magnetic textures, modulate skyrmion topology, and control domain wall motion, in addition to serving as obstacles. Through defect engineering, it is possible to induce, control, and stabilize such behaviors for next-generation spintronic and magnonic devices. Further research should focus on validating the proposed mechanisms under realistic conditions, both computationally and experimentally. Experimental implementations involving pulsed microwave fields or spin-polarized currents can enable direct observations of spin wave interference and skyrmion-guided magnon transport in curved geometries. As a result of such efforts, 3D defect-engineered magnetic systems will remain key components of future wave-based computing architectures that are energy-efficient. By bridging atomistic precision with micromagnetic applicability, this work establishes a methodological and conceptual framework for exploring defect mediated phenomena in multiscale magnetic systems. Spin-based memory, logic, and signal processing technologies can be significantly impacted by anisotropy and topology as shown here, with significant effects on spin dynamics.

## Methods

This study continues the development of the $\mu$-ASD module of UppASD, first introduced in Ref^19^.. This is a specialized simulation package for spin dynamics that connects the atomistic and micromagnetic scale. The $\mu$-ASD module is based on the LLG equation, which governs the temporal evolution of magnetic moments under several interactions, as discussed below. The multiscale nature of the method facilitates the integration of atomistic spin dynamics and micromagnetic simulations, enabling the study of systems where localized atomic-scale phenomena, such as defects, coexist with larger micromagnetic structures like domain walls and skyrmions. The relevant parameters for both scales are derived from the Heisenberg Hamiltonian.

For atomistic interactions, the LLG equation in the form of Landau-Lifshitz (LL), describing the dynamics of an atomic spin is given by:

11 $$\begin{aligned} \frac{\textrm{d} \textbf{m}_i}{\textrm{d}t} = - \frac{\gamma }{1+\alpha ^2}(\textbf{m}_i \times \textbf{H}_i^\textrm{eff} + \frac{\alpha }{m}\textbf{m}_i \times (\textbf{m}_i \times \textbf{H}_i^\textrm{eff})) \end{aligned}$$

where $\gamma$ is the gyromagnetic ratio, $\mathbf {m_i}$ is the magnetic moment of atom *i* and *m* is the modulus of the magnetic moment. A constant Gilbert damping parameter of $\alpha = 0.1$ was used in all simulations reported in this study. The effective field incorporates multiple contributions:

12 $$\begin{aligned} \textbf{H}^\textrm{eff}&= \textbf{H}^{\textrm{exc}} + \textbf{H}^{\textrm{DM}} + \textbf{H}^{\textrm{ani}} + \textbf{H}^{\textrm{Z}} \end{aligned}$$

13 $$\begin{aligned} \textbf{H}_i^{\textrm{exc}}&= -\sum _{j \ne i} J_{ij} \textbf{m}_j \end{aligned}$$

14 $$\begin{aligned} \textbf{H}_i^{\textrm{DM}}&= -\sum _{j \ne i} \textbf{D}_{ij} \times \textbf{m}_j \end{aligned}$$

15 $$\begin{aligned} \textbf{H}_i^{\textrm{ani}}&= -2K_a \textbf{m}_i \end{aligned}$$

16 $$\begin{aligned} \textbf{H}^{\textrm{Z}}&= -g \mu _B \textbf{B}_\textrm{ext} \end{aligned}$$

where $\mu _B$ is the Bohr magneton, *g* is the g-factor, and the different terms in Eq. 12 represents the Heisenberg exchange, the Dzyaloshinskii-Moriya (DM), the anisotropy and external magnetic fields, respectively. These fields are governed by the interaction couplings ${J}_{ij}$ (Heisenberg exchange), $\textbf{D}_{ij}$ (DMI), anisotropy constant $K_a$, and magnetic field $\textbf{B}_\textrm{ext}$. The minus sign in Eqs. (15) and (21) is consistent with the fact that a positive anisotropy constant $K_a$ favors alignment of the atomic magnetic moment or magnetization with the easy axis.

The micromagnetic dynamics is described by the micromagnetic LL differential equation as^40^:

17 $$\begin{aligned} \frac{\partial \textbf{m}}{\partial t} =- \frac{\gamma }{1+\alpha ^2}( \textbf{m} \times \textbf{H}^\textrm{eff} + \alpha \frac{\textbf{m}}{m} \times (\textbf{m} \times {\textbf{H}^\textrm{eff}})) \end{aligned}$$

where $\textbf{m}$ is the magnetization, a continuous function of space and time. The effective field consists of multiple contributions:

18 $$\begin{aligned} \textbf{H}^\textrm{eff}&= \textbf{H}^\textrm{exc} + \textbf{H}^\textrm{DM} + \textbf{H}^\textrm{ani} + \textbf{H}^\textrm{Z} \end{aligned}$$

19 $$\begin{aligned} \textbf{H}^\textrm{exc}&= -\bar{A}_{e} : \nabla \nabla \textbf{m} \end{aligned}$$

20 $$\begin{aligned} \textbf{H}^\textrm{DM}&= -\nabla \cdot \left( \bar{D}_e \times \textbf{m}\right) \end{aligned}$$

21 $$\begin{aligned} \textbf{H}^\textrm{ani}&= -2K_a \textbf{m} \end{aligned}$$

22 $$\begin{aligned} \textbf{H}^\textrm{Z}&= -g\mu _B \textbf{B}_\textrm{ext} \end{aligned}$$

with Eqs. 19-22 representing the exchange field, the DMI contribution, the anisotropy field, and the Zeeman field due to an external magnetic field, respectively. The micromagnetic parameters include the exchange stiffness $\bar{A}_{e}$, DM interaction $\bar{D}_e$, anisotropy constant $K_a$, and external field represented by $\textbf{B}_\textrm{ext}$. The matrix $\bar{A}_e$ is diagonal, while $\bar{D}_e$ is a antisymmetric matrix with zeros on its diagonal elements. In Eq. 19, two tensors are multiplied by the double dot product which results in following expression^41^:

23 $$\begin{aligned} A:B=\sum _{\alpha =1}^3 \sum _{\beta =1}^3 A_{\alpha \beta } B_{ \beta \alpha }. \end{aligned}$$

In making a connection to Eq. 19, we note that in Eq. 23*A* represents $\bar{A}_e$, and *B* represents the operator $\nabla \nabla$.

Atomistic and micromagnetic models differ in their representation of magnetization. Micromagnetics treats magnetization as a continuous field, while atomistic models consider discrete spin moments. To ensure consistency, a mapping between atomistic and micromagnetic exchange parameters is established. The exchange stiffness and DMI tensor in the micromagnetic framework are related to their atomistic counterparts as:

24 $$\begin{aligned} \bar{A}_e&= \frac{1}{2} \sum _{j \ne i} J_{ij} \textbf{r}_{ij}\textbf{r}_{ij} \end{aligned}$$

25 $$\begin{aligned} \bar{D}_e&= \sum _{j \ne i} \textbf{r}_{ij} \textbf{D}_{ij} \end{aligned}$$

26 $$\begin{aligned} \textbf{r}_{ij}&= \textbf{r}_{j} - \textbf{r}_{i} \end{aligned}$$

where $\textbf{r}_{ij}$ is the vector connecting atom *i* with atom *j*. In materials with spatially varying exchange coefficients, micromagnetic parameters are computed separately for different atomic environments and averaged accordingly to ensure consistency across scales. This multiscale approach enables accurate modeling of magnetic materials with heterogeneous exchange interactions and DMI anisotropies.

In multiscale simulations one integrates the atomistic and continuum domains, where each region follows its respective governing equations. To facilitate connection between these regions, a specialized technique is employed to solve the relevant differential equations. A smooth transition at the interface is ensured by introducing padding atoms and padding nodes within their respective domains. Unlike conventional magnetization formulations, these padding elements acquire their magnetic moments through interpolation from their respective domains, thereby maintaining smoothness at the atomistic-continuum interface. Additionally, within the atomistic region, coarse-graining techniques may be applied near the continuum interface, leading to the formation of coarse-graining and damping bands to enhance computational efficiency and stability^40,42^.

In this study, the micromagnetic system under investigation is an iron-iridium (Fe-Ir) composite. This system is characterized by nearest neighbor atomic interactions, with an exchange interaction value of 0.419 mRy, a Dzyaloshinskii-Moriya interaction (DMI) of 0.132 mRy, and a uniaxial anisotropy of 0.059 mRy. These values have been taken from experimental measurements as it was reported in Ref^18^.. The lattice constant $a = 0.38~\textrm{nm}$was taken from Ref^43^.. We note that the nearest neighbor approximation adopted here for the atomistic region is not a limitation of the formalism, presented above, but rather a practical choice for the presently investigated system. In all simulations presented here, we consider a larger micromagnetic framework with an atomistic region in the middle, enabling a high-resolution analysis of atomic-scale phenomena. The corresponding micromagnetic parameters when calculated from Eqs. 24-25, will have $A_{e}$ with a value of 0.627 mRy/Å, ${D}_e$ with a value of 0.099 mRy/Å^2^, and the anisotropy field, which is the same as for the atomistic region, as it represents a local field. These parameters were kept the same for all simulations presented here, except for smaller regions in the atomic domain, where we in some cases considered local impurities with different magnetic anisotropy.

A primary focus of this study is the introduction of a localized defect within the atomistic region. By embedding such a small-scale defect into the significantly larger micromagnetic system, the aim is to investigate how the defect influences the overall magnetic behavior and dynamics of the system. This study particularly emphasizes understanding the interaction of the defect with specific magnetic configurations, namely domain walls and skyrmions. These configurations are chosen due to their fundamental importance in spintronics and their sensitivity to local perturbations. In addition, this allows to study the dynamics of magnetic objects with trivial and non-trivial topology. The introduction of defects in the middle of the system provides an ideal platform to examine how localized structural or magnetic variations can alter domain wall motion, skyrmion lifetime, and overall magnetic properties.

In this study, the calculation of the skyrmion topological charge in 3D micromagnetic simulations has been successfully implemented within the computational framework. This enhancement extends conventional 2D skyrmion charge calculations to fully 3D systems, allowing for a more general characterization of skyrmion tubes, and other 3D topological textures^7,10,44,45^. In the micromagnetic continuum limit, the topological skyrmion number in 2D is defined as:

27 $$\begin{aligned} Q^S = \frac{1}{4\pi } \int \textbf{m} \cdot \left( \frac{\partial \textbf{m}}{\partial x} \times \frac{\partial \textbf{m}}{\partial y} \right) d^2r \end{aligned}$$

where $\textbf{m}$ is the normalized magnetization vector. This equation is typically valid in 2D micromagnetic systems, where the skyrmion charge describes the number of times the magnetization field wraps around the unit sphere^6,9^. However, for fully 3D skyrmion tubes, additional generalizations are required, incorporating spatial variations along the third dimension^46,47^. The volumetric skyrmion number in 3D is defined as^9,48,49^:

28 $$\begin{aligned} Q^V = \frac{1}{8\pi } \int \textbf{m} \cdot \left( \frac{\partial \textbf{m}}{\partial x} \times \frac{\partial \textbf{m}}{\partial y} \right) d^3r. \end{aligned}$$

It is worth to mention here that the integration in Eq. 28 extends over the volume $V$ of the magnetic region where the topological texture exists. Because the integrand has dimensions of $\text {1/[L]}^2$ where L is the length, the integral over volume introduces units of length, giving $Q^V$ units of length. Therefore, Eq. (28) is dimensionally consistent and does not represent a conventional topological charge (which is dimensionless) but rather a measure of the spatial extent of the topological density within the three-dimensional volume. We use the notation $\bar{Q}^V= \frac{Q^V}{L}$ to indicate the dimensionless 3D skyrmion number. By generating a 3D skyrmion, the computed volumetric skyrmion number is confirmed to be $\bar{Q}^V = 1$, consistent with the expected topological invariant for an isolated skyrmion. This implementation enables further investigations into 3D skyrmion dynamics and expanding the micromagnetic simulations in topological magnetism.

This work aims to uncover the fundamental mechanisms by which defects in hybrid micromagnetic-atomistic systems impact the stability, dynamics, and structure of magnetic configurations. To achieve this, the defect is treated at an atomistic level, providing a high-resolution view of its interactions and influence. The surrounding regions, which are free of defects, are modeled using a micromagnetic approach. This hybrid modeling approach enables a multiscale analysis, combining the benefits of atomistic precision in the defect region with the computational efficiency of micromagnetic simulations for the larger system.

The study comprises two distinct series of simulations, each designed to probe the response of the system under different initial magnetic configurations. In the first series, a domain wall is introduced within the micromagnetic structure. The interaction of the domain wall with the atomistic defect region is analyzed to elucidate the impact of the defect on domain wall dynamics, structure, and stability. In the second series, the system is initialized with a 3D skyrmion, a topologically protected magnetic structure. Here, the focus is on understanding how the defect alters the skyrmion properties, such as its size, lifetime, and dynamics.

This dual-approach methodology provides a comprehensive understanding of defect-induced effects in micromagnetic systems. It not only sheds light on the fundamental mechanisms governing defect interactions in hybrid micromagnetic-atomistic systems but also has potential implications for designing advanced magnetic materials and devices where precise defect engineering plays a crucial role.

## Supplementary Information

Supplementary Information 1.

## Data Availability

All data generated or analysed during this study are included in this published article [and its supplementary information files].
