# Supplementary material for: Hybrid micromagnetic and atomistic modeling of magnetization dynamics induced by engineered defects
Source: Sci Rep. 2025 Dec 21;15:44232. doi: 10.1038/s41598-025-31866-6 (PMC12722306; doi:10.1038/s41598-025-31866-6)
Supplement: Supplementary file 1 — Supplementary Information 1. [file 41598_2025_31866_MOESM1_ESM.zip › Revised-Supplementary/Supplementary Figure Legends.pdf]

Supplementary Figure Legends

Fig. S1: (Color online) Representation of the components of the atomic magnetic moment of atom i in spherical and Cartesian coordinates.
Fig. S2: (Color online) Schematic representation of a spherical wave interference pattern provided by 2 point wave sources shown in red. Far from the sources, in the central region the wave vector $\vec{k}$ can be approximated to be aligned along the y direction as shown by the red arrow which is the sum of the individual wave vectors (in blue and green color) produced by the red sources, respectively.
Fig. S3: (Color online) Schematic representation of the different vectors and parameter used to describe the propagation of the spin waves. The symbol d represents the distance between the slits while η accounts for the distance between the reference frame and the first slit. The vectors are described by the red and blue arrows.
Fig. S4: 1D domain wall indicating the angles θ, ϕ and the domain wall width Δ.
Fig. S5: Reference frame used to define the angles θ and φ of the magnetization in spherical coordinates.
Fig. S6: 3D Skyrmion motion by applying STT of 15 m/s through the simulation cell, with a defect region shaped as a tetrahedron cluster with uniaxial anisotropy and a) hard axis along z-direction, b) easy axis along z-direction with strength of 0.11 mRy. The color bar is showing the z component of normalized magnetization.
Fig. S7: 3D Skyrmion motion by applying STT of 25 m/s through the simulation cell, with a defect region shaped as a tetrahedron cluster with uniaxial anisotropy, a) hard axis along z-direction and b) easy axis along z-direction with strength of 0.9 mRy. The color bar is showing the z component of normalized magnetization.
