# Supplementary material for: Hybrid micromagnetic and atomistic modeling of magnetization dynamics induced by engineered defects
Source: Sci Rep. 2025 Dec 21;15:44232. doi: 10.1038/s41598-025-31866-6 (PMC12722306; doi:10.1038/s41598-025-31866-6)
Supplement: Supplementary file 1 — Supplementary Information 1. [file 41598_2025_31866_MOESM1_ESM.zip › Revised-Supplementary/Supplementary.pdf]

Micromagnetic-atomistic hybrid modeling of defect-induced magnetization dynamics
—Supplementary—

Nastaran Salehi[1], Olle Eriksson[1,2], Johan Hellsvik[3], and Manuel Pereiro[1]
[1]*Department of Physics and Astronomy, Uppsala University, 751 21 Uppsala, Sweden*
[2]*WISE - Wallenberg Initiative Materials Science for Sustainability, Department of Physics and Astronomy, Uppsala University, SE-751 20 Uppsala, Sweden*
[3]*PDC Center for High Performance Computing, KTH Royal Institute of Technology, SE-100 44 Stockholm, Sweden*

1 Magnon dispersion relation

Starting with a Hamiltonian that describes the fundamental interactions of the Fe-Ir system given by:

$$\mathscr{H} = -\frac{1}{2}\sum_{ij}\mathbf{s}_i\mathscr{J}_{ij}\mathbf{s}_j = -\frac{1}{2}\sum_{ij}\left(J_{ij}\mathbf{s}_i\mathbf{s}_j + \mathbf{D}_{ij}\mathbf{s}_i\times s_j\right) \tag{1}$$

where:

$$\mathscr{J}_{ij} = \begin{pmatrix} J_{ij} & D^z_{ij} & -D^y_{ij} \\ -D^z_{ij} & J_{ij} & D^x_{ij} \\ D^y_{ij} & -D^x_{ij} & J_{ij} \end{pmatrix} \tag{2}$$

The exchange interaction between atom i with atomic magnetic moment s_i and atom j with atomic magnetic moment s_j is represented by J_{ij} while the components of the Dzyaloshinskii-Moriya (DM) interaction are given by D^α_{ij}. The equation of motion for the atomic magnetic moments is given by:

$$-i\hbar\frac{\mathrm{d}\mathbf{s}_k}{\mathrm{d}t} = [\mathscr{H},\mathbf{s}_k] = -\frac{1}{2}\left(\left[\sum_{ij}J_{ij}\mathbf{s}_i\mathbf{s}_j,\mathbf{s}_k\right] + \left[\sum_{ij}\mathbf{D}_{ij}\mathbf{s}_i\times\mathbf{s}_j,\mathbf{s}_k\right]\right) \tag{3}$$

If we recast Eq. 3 in components, then:

$$-i\hbar\frac{\mathrm{d}s_{k\eta}}{\mathrm{d}t} = -\frac{1}{2}\left(\left[\sum_{ij}J_{ij}s_{i\alpha}s^\alpha_j, s_{k\eta}\right] + \left[\sum_{ij}D^\alpha_{ij}\varepsilon_{\alpha\beta\gamma}s^\beta_i s^\gamma_j, s_{k\eta}\right]\right) \tag{4}$$

As notation, we use the latin letter for atomic positions while the greek letters are used for the components of the vector or tensor. By using the commutation relations of the spins:

$$\begin{aligned}\left[s_{k\eta}, s_{i\alpha}\right] &= -ig\mu_B\delta_{ki}\varepsilon_{\eta\alpha\gamma}s^\gamma_k \\ \left[s_{k\eta}, s^\alpha_j\right] &= -ig\mu_B\delta_{kj}\varepsilon^\alpha_{\eta\gamma}s^\gamma_k,\end{aligned} \tag{5}$$

and after some algebra, the different terms of the equation of motion are given by:

$$\begin{aligned}\left[\sum_{ij}J_{ij}\mathbf{s}_i\mathbf{s}_j,\mathbf{s}_k\right] &= 2ig\mu_B\sum_{j\neq k}J_{kj}\varepsilon_{\eta\alpha\gamma}s^\gamma_k s^\alpha_j \\ \left[\sum_{ij}D^\alpha_{ij}\varepsilon_{\alpha\beta\gamma}s^\beta_i s^\gamma_j, s_{k\eta}\right] &= 2ig\mu_B\sum_{j\neq k}D^\alpha_{kj}\varepsilon_{\alpha\beta\gamma}\varepsilon^\gamma_{\eta\chi}s^\chi_k s^\beta_j,\end{aligned} \tag{6}$$

Assuming the atomic magnetic moment of the system is in the ground state with the moments pointing almost along the z direction, i.e., $\mathbf{s}_i = s_i\hat{\mathbf{e}}_3$ with $\hat{\mathbf{e}}_3$ being a orthogonal unit vector as indicated in Fig. S1. We perturb the ground-state by using a spin-spiral excitation, and so the magnetic moment vector can be recasted in spherical coordinates like:

$$\mathbf{s}_i = s_i\left(\sin\theta_i\cos\phi_i\hat{\mathbf{e}}_1 + \sin\theta_i\sin(c\phi_i)\hat{\mathbf{e}}_2 + \cos(\theta_i)\hat{\mathbf{e}}_3\right) \tag{7}$$

where $\hat{\mathbf{e}}_1$ and $\hat{\mathbf{e}}_2$ are orthogonal unit vectors as shown in Fig. S1 and $c = \pm 1$ represents the chirality of the spin waves.

Keeping in mind that we are disregarding Stoner excitations, in which the length of the magnetic moment is considered constant as time evolves, we obtain, after some algebra, the equation of motion:

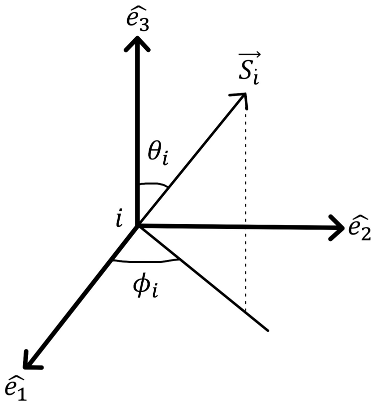

FIG. S1. (Color online) Representation of the components of the atomic magnetic moment of atom i in spherical and Cartesian coordinates.

$$
\begin{aligned}
\frac{\mathrm{d}s_i}{\mathrm{d}t} &= 0 \\
c\sin\theta_i\frac{\mathrm{d}\phi_i}{\mathrm{d}t} &= \frac{g\mu_B}{\hbar}\sum_{j\neq i} s_j[J_{ij}(-\cos\theta_i\sin\theta_j\cos(\phi_i-\phi_j)+\sin\theta_i\cos\theta_j)-(\mathbf{D}_{ij}\hat{\mathbf{e}}_1)\sin\theta_i\sin\theta_j\sin(c\phi_j) \\
&\quad -(\mathbf{D}_{ij}\hat{\mathbf{e}}_1)\sin(c\phi_i)\cos\theta_i\cos\theta_j+(\mathbf{D}_{ij}\hat{\mathbf{e}}_2)\sin(\theta_i)\sin\theta_j\cos\phi_j \\
&\quad +(\mathbf{D}_{ij}\hat{\mathbf{e}}_2)\cos(\phi_i)\cos\theta_i\cos\theta_j+(\mathbf{D}_{ij}\hat{\mathbf{e}}_3)\cos(\theta_i)\sin\theta_j\sin(c(\phi_i-\phi_j)] \\
\frac{\mathrm{d}\phi_i}{\mathrm{d}t} &= \frac{-g\mu_B}{\hbar}\sum_{j\neq i} s_j[J_{ij}\sin\theta_j\sin(c(\phi_i-\phi_j))-(\mathbf{D}_{ij}\hat{\mathbf{e}}_1)\cos\phi_i\cos\theta_j-(\mathbf{D}_{ij}\hat{\mathbf{e}}_2)\sin(c\phi_i)\cos\theta_j \\
&\quad +(\mathbf{D}_{ij}\hat{\mathbf{e}}_3)\sin\theta_j\cos(\phi_i-\phi_j)]
\end{aligned}
\tag{8}
$$

Far from the point sources that produce the magnetic perturbation, we can assume that θ_i is constant and consequently, $\frac{\mathrm{d}\theta_i}{\mathrm{d}t}=0$. Then, in Eq. 8, by summing up the second equation with the third one multiplied by $ic\cos\theta_i\frac{\mathrm{d}\theta_i}{\mathrm{d}t}$ and by time-averaging it, we end up in the following expression:

$$
\theta_i\frac{\mathrm{d}(c\phi_i)}{\mathrm{d}t} = \frac{g\mu_B}{\hbar}\sum_{j\neq i} s_j[-J_{ij}(\theta_j e^{i(\phi_i-\phi_j)}-\theta_i)-ic(\mathbf{D}_{ij}\hat{\mathbf{e}}_3)\theta_j e^{i(\phi_i-\phi_j)}] \tag{9}
$$

The Fourier transform of Eq. 9 is:

$$
\theta_\mu\omega^c = \frac{g\mu_B}{\hbar}\sum_{[\nu+\mathbf{R}]\neq\mu} s_{[\nu+\mathbf{R}]}[-J_{[\nu+\mathbf{R}]}(\theta_{[\nu+\mathbf{R}]}e^{i(q(\tau_\mu-\tau_\nu-\mathbf{R})+\phi_\mu-\phi_\nu)}-\theta_\mu)-ic(\mathbf{D}_{\mu[\nu+\mathbf{R}]}\hat{\mathbf{e}}_3)\theta_{[\nu+\mathbf{R}]}e^{i(q(\tau_\mu-\tau_\nu-\mathbf{R})+\phi_\mu-\phi_\nu)}] \tag{10}
$$

where the following notation has been used: $\mathbf{R}_i=\tau_\mu$ (atomic position of atom i), $\mathbf{R}_j=\mathbf{R}+\tau_\mu$ (atomic position of atom j and $\mathbf{R}$ is the lattice displacement), $\phi_i=\omega t+\mathbf{q}\tau_\mu+\phi_\mu$ (phase angle of atom i), $\phi_j=\omega t+\mathbf{q}\tau_\nu+\phi_\nu$ (phase angle of atom j), $\phi_i-\phi_j=\mathrm{q}(\tau_\mu-\mathbf{R})+\phi_\mu-\phi_\nu$ (phase difference between atom i and j, respectively), $i\to\mu$, $j\to[\nu+\mathbf{R}]$. Moreover, we also use that $\frac{\mathrm{d}(c\phi_i)}{\mathrm{d}t}=\omega_i^c=\text{constant}=\omega^c\ \forall i$. By following this change of notation, from now on, the greek letters μ and ν will refer also to atomic positions rather than vector coordinates. In Eq. 9, we have also Fourier transformed the coupling interactions as given by:

$$
\begin{aligned}
J_{\mu\nu}(\mathbf{q}) &= \delta_{\mu\nu}J_{\mu\mu}-\sum_{\mathbf{R}}J_{\mu[\nu+\mathbf{R}]}e^{i(q(\tau_\mu-\tau_\nu-\mathbf{R})} \\
\mathbf{D}_{\mu\nu} &= \delta_{\mu\nu}\mathbf{D}_{\mu\mu}-\sum_{\mathbf{R}}\mathbf{D}_{\mu[\nu+\mathbf{R}]}e^{i(q(\tau_\mu-\tau_\nu-\mathbf{R})}
\end{aligned}
\tag{11}
$$

In the ansatz of the current formulation, it follows that:

$$
s_{[\nu+\mathbf{R}]}=s_\nu;\ \theta_{[\nu+\mathbf{R}]}=\theta_\nu \tag{12}
$$

Then, Eq. 9 can be recasted as:

$$\tilde{\theta}_\mu \hbar\omega^c = g\mu_B \sum_{\nu\neq\mu} \sqrt{s_\mu}\sqrt{s_\nu}\tilde{\theta}_\nu [J_{\mu\nu}(\mathbf{q}) - \delta_{\mu\nu}\sum_\lambda \frac{s_\lambda}{s_\mu} J_{\mu\lambda}(\mathbf{q}=0) + ic(\mathbf{D}_{\mu\nu}(\mathbf{q})\hat{\mathbf{e}}_3)] \tag{13}$$

where we defined $\tilde{\theta}_\mu = \sqrt{s_\mu}\theta_\mu e^{-i\phi_\mu}$. If we define

$$\tilde{T}_{\mu\nu}(\mathbf{q}) = J_{\mu\nu}(\mathbf{q}) - \delta_{\mu\nu}\sum_\lambda \frac{s_\lambda}{s_\mu} J_{\mu\lambda}(\mathbf{q}=0) + ic(\mathbf{D}_{\mu\nu}(\mathbf{q})\hat{\mathbf{e}}_3) \tag{14}$$

the final from of Eq. 13 can be given by:

$$\tilde{\theta}_\mu \hbar\omega^c = g\mu_B \sum_{\nu\neq\mu} \sqrt{s_\mu}\sqrt{s_\nu}\tilde{T}_{\mu\nu}(\mathbf{q})\tilde{\theta}_\nu \tag{15}$$

By solving the eigenvalue problem described by Eq. 15, it is only required to diagonalise the following tensor:

$$\mathscr{A}_{\mu\nu} = \frac{g\mu_B}{\hbar} \sum_{\nu\neq\mu} \sqrt{s_\mu}\sqrt{s_\nu}\tilde{T}_{\mu\nu}(\mathbf{q}) \tag{16}$$

The eigenvalues of $\mathscr{A}_{\mu\nu}$ matrix provide the adiabatic magnon spectra as a function of the wave vector in the reciprocal space.

Assuming that the magnetic texture of the material have a small angle with respect to the $\hat{\mathbf{e}}_3$ direction, we can assume that $\tilde{\theta}_\mu \approx \tilde{\theta}_\nu$. Moreover, for simplicity without loss of generality we assume $s_\mu = s_\nu = s$. Then, Eq. 15 can be recasted as:

$$\hbar\omega^c = g\mu_B s \sum_{\nu\neq\mu} \tilde{T}_{\mu\nu}(\mathbf{q}) \tag{17}$$

For small $\mathbf{q}$-vector and considering that $\mu \neq \nu$, the exchange part of the magnon energies can be reduced to:

$$\Re(J_{\mu\nu}(\mathbf{q})) \simeq \frac{1}{2}\sum_{\mathbf{R}} J_{\mu[\nu+\mathbf{R}]}\mathbf{q}^2(\tau_\mu - \tau_\nu - \mathbf{R})^2 \tag{18}$$

Considering that the exchange interaction is relevant for only first neighbouring atoms and all atoms present the same interaction (J):

$$\Re(J_{\mu\nu}(\mathbf{q})) \simeq \frac{J}{2} z a^2 q^2 \tag{19}$$

where z is coordination number and a is the lattice constant and the dispersion relation for the exchange part of the hamiltonian is given by:

$$\hbar\omega = \frac{1}{2} g\mu_B s J (za)^2 q^2 \tag{20}$$

where $D_{ex} = \frac{1}{2} g\mu_B s J(za)^2$ represents the spin-wave stiffness.

For the DM interaction with small $\mathbf{q}$, we have:

$$\Re(\mathbf{D}_{\mu\nu}) = -\sum_{\mathbf{R}} c\mathbf{D}^z_{\mu[\nu+\mathbf{R}]}\left[\mathbf{q}(\tau_\mu - \tau_\nu - \mathbf{R})\right] \tag{21}$$

Assuming as in the case of the exchange interaction, that DM interaction only is relevant for the first-neighbours and it has the same strength (D) as it is the case for the current system considered in this article,

$$\Re(\mathbf{D}_{\mu\nu}) = -cDq\sum_{\mathbf{R}} \mathbf{D}^z_{\mu[\nu+\mathbf{R}]}|\tau_\mu - \tau_\nu - \mathbf{R}|\cos\theta(\mathbf{q},\mathbf{R}) \tag{22}$$

For the simple cubic case, $\mathbf{R} = \{a(\pm1,0,0), a(0,\pm1,0), a(0,0,\pm1)\}$, the DM term can be finally recasted in:

$$\Re(\mathbf{D}_{\mu\nu}) = -2cDa(q^x + q^y + q^z) \tag{23}$$

As shown in Fig. S2, for a spherical wave pattern provided by 2 point wave sources far from the sources, the total wave vector in the central region almost only the x component survives, so that we can approximate the DM term by:

$$\Re(\mathbf{D}_{\mu\nu}) \simeq -2cDaq \tag{24}$$

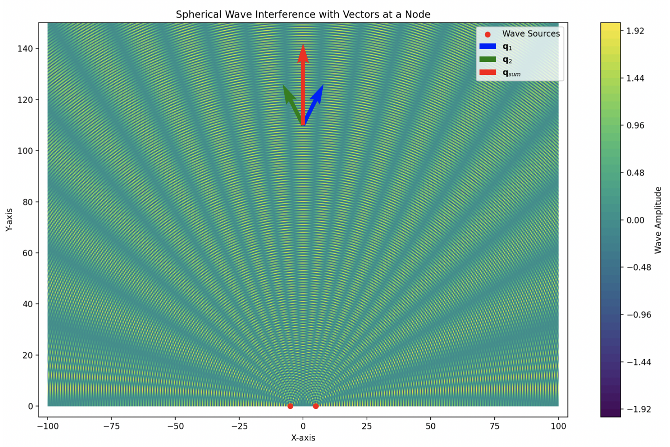

FIG. S2. (Color online) Schematic representation of a spherical wave interference pattern provided by 2 point wave sources shown in red. Far from the sources, in the central region the wave vector $\vec{k}$ can be approximated to be aligned along the y direction as shown by the red arrow which is the sum of the individual wave vectors (in blue and green color) produced by the red sources, respectively.

In conclusion the dispersion relation for the hamiltonian given in Eq. 1 can be recasted in the following expression:

$$\hbar\omega^c = D_{ex}q^2 - cD_{dm}q \tag{25}$$

where $D_{dm} = g\mu_B sDz^2 a$.

Finally, the wave vector can be given by:

$$q = \frac{cD_{dm} \pm \sqrt{D_{dm}^2 \mp 4D_{ex}\hbar\omega}}{2D_{ex}} \tag{26}$$

2 Model on the diffraction pattern of the macromagnetic spin waves

A spherical wave, generated by two point sources whose intensity decays exponentially, can be described mathematically using the following expression:

$$\mathbf{W}_{ext} = W_1 \frac{e^{i(q|\mathbf{r}-\mathbf{r}_1|-\omega t)}}{|\mathbf{r}-\mathbf{r}_1|} e^{|\mathbf{r}-\mathbf{r}_1|\xi} + W_2 \frac{e^{i(q|\mathbf{r}-\mathbf{r}_2|-\omega t)}}{|\mathbf{r}-\mathbf{r}_2|} e^{|\mathbf{r}-\mathbf{r}_2|\xi} \tag{27}$$

where ξ is the attenuation coefficient induced by the medium where the spin waves propagate and W_1 and W_2 are the amplitudes of the spherical waves created in slits 1 and 2, repectively. The definition of the vectors and notation of the parameters used on this section is shown in Fig. S3. The intensity of the wave function is obtaining by multiplying the wave function by the complex conjugate, so that:

$$|W_{ext}|^2 = W_0^2 \left[\frac{e^{2|\mathbf{r}-\mathbf{r}_1|\xi}}{|\mathbf{r}-\mathbf{r}_1|^2} + \frac{e^{2|\mathbf{r}-\mathbf{r}_2|\xi}}{|\mathbf{r}-\mathbf{r}_2|^2} + \frac{2\cos(q(|\mathbf{r}-\mathbf{r}_1| - |\mathbf{r}-\mathbf{r}_2|))e^{(|\mathbf{r}-\mathbf{r}_1|+|\mathbf{r}-\mathbf{r}_2|)\xi}}{|\mathbf{r}-\mathbf{r}_1||\mathbf{r}-\mathbf{r}_2|} \right] \tag{28}$$

where, for simplicity, we have assumed that $W_1 = W_2 = W_0$. Following the setup and the reference frame shown in Fig. S3, the coordinates of the vectors are given by: $\mathbf{r}_1 = (0, d+\eta)$, $\mathbf{r}_2 = (0,\eta)$, $\mathbf{r} = (x,y)$, $\mathbf{r}-\mathbf{r}_1 = (x, y-(d+\eta))$ and $\mathbf{r}-\mathbf{r}_2 = (x, y-\eta)$. The final expression of the wave amplitude in the reference frame pictured in Fig. S3 is:

$$\begin{aligned} |W_{ext}|^2 = W_0^2 \Bigg[& \frac{e^{2\sqrt{x^2+(y-(d+\eta))^2}\xi}}{x^2+(y-(d+\eta))^2} + \frac{e^{2\sqrt{x^2+(y-\eta)^2}\xi}}{x^2+(y-\eta)^2} \\ & + \frac{2\cos(q(\sqrt{x^2+(y-(d+\eta))^2} - \sqrt{x^2+(y-\eta)^2}))e^{(\sqrt{x^2+(y-(d+\eta))^2}+\sqrt{x^2+(y-\eta)^2})\xi}}{\sqrt{x^2+(y-(d+\eta))^2}\sqrt{x^2+(y-\eta)^2}} \Bigg] \end{aligned} \tag{29}$$

Finally, for small angle deviations we can approximate the average angle deviation squared by:

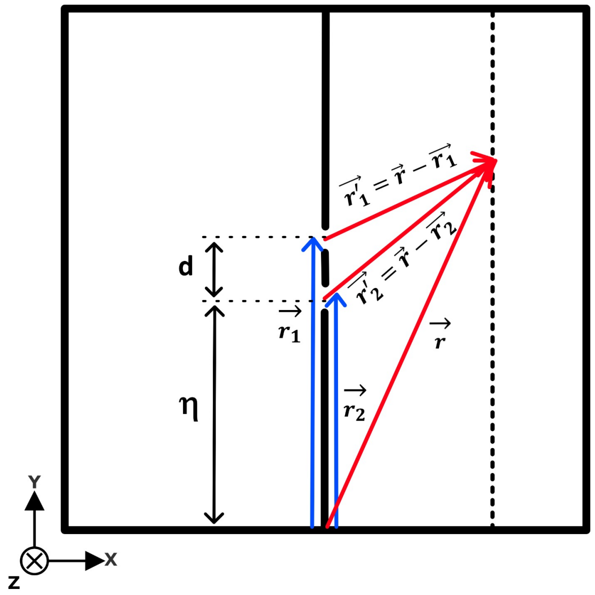

FIG. S3. (Color online) Schematic representation of the different vectors and parameter used to describe the propagation of the spin waves. The symbol d represents the distance between the slits while η accounts for the distance between the reference frame and the first slit. The vectors are described by the red and blue arrows.

$$\langle\theta\rangle^2 \simeq \langle\frac{\gamma W_{ext}}{\omega}\rangle^2 + \sigma = \left(\frac{\gamma}{\omega}\right)^2 \langle W_{ext}\rangle^2 + \sigma \tag{30}$$

where σ represents background intensity, potentially arising from microwave field-driven energy input. The attenuation coefficient ξ is the imaginary part of the frequency of the spin wave and it is related to the Gilbert damping α by:

$$\xi = \frac{\alpha\omega}{v_g} \tag{31}$$

where v_g is the group velocity and it is defined as $\mathbf{v}_g = \frac{\partial\omega}{\partial\mathbf{q}}$. By using Eq. 25, the attenuation coefficient can finally be derived as:

$$\xi^c = \frac{\alpha\omega}{2\frac{D_{ex}}{\hbar}q - c\frac{D_{dm}}{\hbar}} \tag{32}$$

The parameters used in the spin wave model to predict the form of the diffraction pattern are collected in Table S1.

3 Theoretical one-dimensional (1D) model for domain wall acceleration after geometric constriction by the slit

The double slit imposes a geometric constraint, forcing the DW to deform. This deformation stores potential energy, modelled by the term $V(q)$. We assume $V(q)$ has a maximum value V_0 at the slit position q', representing the elastic energy stored due to compression against exchange and anisotropy forces. The potential can be mathematically modeled by a smooth Dirac delta in spatial dimension by using the following expression:

$$V(q-q\prime) = V_0\, e^{-\frac{q-q\prime}{a}} \tag{33}$$

where a is the width of the potential.

We now consider a 1D model of the DW dynamics subject to the action of a field H perpendicular to the x-axis and aligned along the z-direction using the reference frame depicted in Fig. S4. The magnetization is represented in spherical coordinates as indicated in Fig. S5. The one-dimensional domain wall is fully characterized by three parameters: $q(x,t)$ specifies the wall center position along the x-axis, $\Delta(x,t)$ determines the domain wall width, and $\phi(x,t)$ defines the magnetization rotation angle. The resulting magnetization profile is described everywhere by the function:

Quantity	Magnitude	unit
η	80	Å
d	20	Å
x	82	Å
ξ	-0.561918	$Å^{-1}$
α	0.1	
f	1	10^{12} Hz
γ	176085.81	$10^6 \frac{Hz}{T}$
z	6	
q	-1.52116	$Å^{-1}$
D	0.28774338	10^{-21} J
J	0.91336725	10^{-21} J
σ	0.0254	degree^2
W_0	44800	degree · T
s	2.23	μ_B

Table S1. Table of parameters used in the spin wave model to describe the diffraction pattern.

$$\theta(x,t) = 2\tan^{-1}\left[\exp\left(\frac{x-q(t)}{\Delta(t)}\right)\right] \tag{34}$$

$$\varphi(x,t) = \phi(t) \tag{35}$$

where $\varphi(x,t)$ is the angle between the y-axis and the plane formed by the magnetization vector and the projection of the magnetization over the y-z plane as shown in Fig. S5. For simplicity, it is assumed to be independent of the position and only can change in time.

In order to obtain the equations of motion of the magnetization, and in particular, the velocity of the domain wall, we will start from the Lagrangian form of the micromagnetic dynamic equations:

$$\frac{\partial L}{\partial \zeta} - \frac{\mathrm{d}}{\mathrm{dt}}\left(\frac{\partial L}{\partial \dot{\zeta}}\right) + \frac{\partial F}{\partial \dot{\zeta}} = 0 \tag{36}$$

where ζ is the generalized coordinate and can be represented by q, Δ or ϕ. The Lagrange function is $\mathscr{L} = \int_0^\infty \left(\mathscr{E} + \frac{M_s}{\gamma}\dot{\phi}\cos\theta\right) dx - V(q-q')$ and the dissipation energy is $\mathscr{F} = \left(\frac{\alpha M_s}{2\gamma}\right)\int_0^\infty \left(\dot{\theta}^2 + \dot{\phi}^2\sin^2\theta\right) dx$ (1). Moreover, $\mathscr{E}$ is the micromagnetic density energy, γ is the gyromagnetic ratio, α is the Gilbert damping and M_s is the saturation magnetization. The micromagnetic energy density for a 1D domain wall model, considering contributions from exchange (A), Dzyaloshinskii-Moriya interaction (D), uniaxial anisotropy (K_0), second-order uniaxial transverse anisotropy (K), and an external magnetic field (H), is given by:

$$\mathscr{E} = A\left(\frac{\partial\theta}{\partial x}\right)^2 + \frac{D}{\Delta}\sin\phi\sin\theta + K_0\sin^2\theta + K\sin^2\theta\sin^2\phi - \mu_0 M_s H\cos\theta \tag{37}$$

where μ_0 is the vacuum magnetic permeability. Thus, the space-integrated Lagrangian $\mathscr{L}$ can be recasted in the following form:

$$\mathscr{L} = \frac{2A}{\Delta} + \frac{\pi D}{2\Delta}\sin\phi + 2\Delta\left(K_0 + K\sin^2\phi\right) - 2\mu_0 M_s H q + \frac{2\mu_0 M_s q}{\gamma}\dot{\phi} - V_0 e^{-\left(\frac{q-q'}{a}\right)^2} \tag{38}$$

Similarly, the space-integrated dissipation function $\mathscr{F}$ takes the form:

$$\mathscr{F} = \frac{\alpha\mu_0 M_s}{\gamma}\left[\Delta\dot{\phi}^2 + \frac{\dot{q}^2}{\Delta} + \frac{\dot{q}\dot{\Delta}}{\Delta}\ln 2 + \frac{\pi^2}{24}\left(\frac{\dot{\Delta}}{\Delta}\right)^2\right] \tag{39}$$

Since we are just interested in the velocity of the domain wall, by setting $\zeta = \phi$ and $\zeta = q$ in the Euler-Lagrange equations

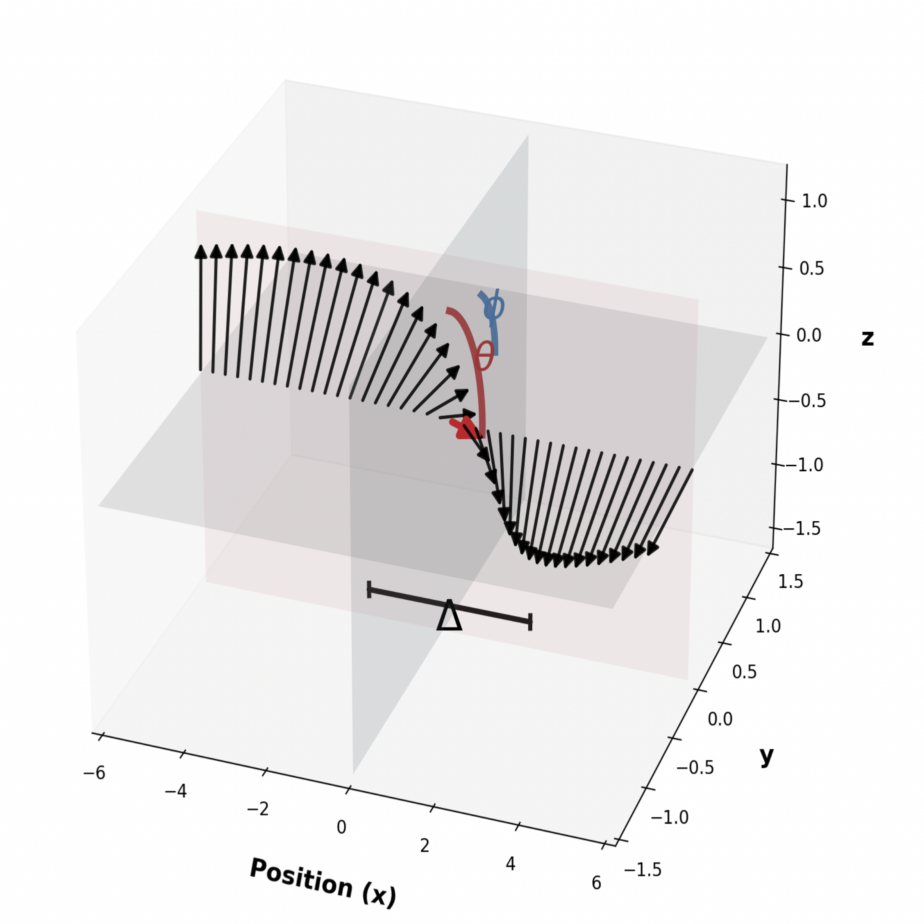

FIG. S4. 1D domain wall indicating the angles θ, ϕ and the domain wall width Δ.

(Eq. 36), we end up with the following equations of motion:

$$\frac{\dot{q}}{\Delta} - \alpha\dot{\phi} = \frac{\pi D \gamma}{4\Delta^2 \mu_0 M_s} \cos\phi + \gamma H_k \sin(2\phi) \tag{40}$$

$$-\gamma H + \dot{\phi} + \eta e^{-\left(\frac{q-q'}{a}\right)^2}(q-q') + \alpha\left(\frac{\dot{q}}{\Delta} + \frac{\dot{\Delta}}{\Delta}\ln\sqrt{2}\right) = 0 \tag{41}$$

where $H_k = \frac{K}{\mu_0 M_s}$ is the uniaxial anisotropy field and $\eta = \frac{\gamma V_0}{\mu_0 M_s a^2}$. Eliminating $\dot{\phi}$ from Eqs. 40-41, the velocity of the domain wall ($\dot{q}$) is finally given by:

$$\dot{q} = \frac{1}{1+\alpha^2}\left(\alpha\gamma\Delta H - \alpha\Delta\eta e^{-\left(\frac{q-q'}{a}\right)^2}(q-q') - \alpha^2\dot{\Delta}\ln\sqrt{2} + \frac{\pi D\gamma}{4\Delta\mu_0 M_s}\cos\phi + \gamma\Delta H_k \sin(2\phi)\right) \tag{42}$$

The chirality induced by the Dzyaloshinskii-Moriya interaction (DMI) affects the domain wall velocity, with the direction of the effect determined by the sign of the DMI constant, D. Specifically, positive D tends to increase velocity, while negative D tends to decrease it. However, focusing on the interaction with the slit potential, the primary mechanism for velocity increase upon traversing the slit involves the second and third terms of Eq. 42. The second term, originating from the slit potential gradient, initially accelerates the domain wall as it approaches the slit ($q-q' < 0$), thereby yielding a positive contribution to the driving force. Although this acceleration is followed by a deceleration after the slit ($q-q' > 0$, yielding a negative contribution), the initial acceleration phase critically modifies the domain wall structure. It induces an abrupt decrease in the domain wall width (Δ), resulting in a negative time derivative ($\dot{\Delta} < 0$). Consequently, the third term becomes positive, providing

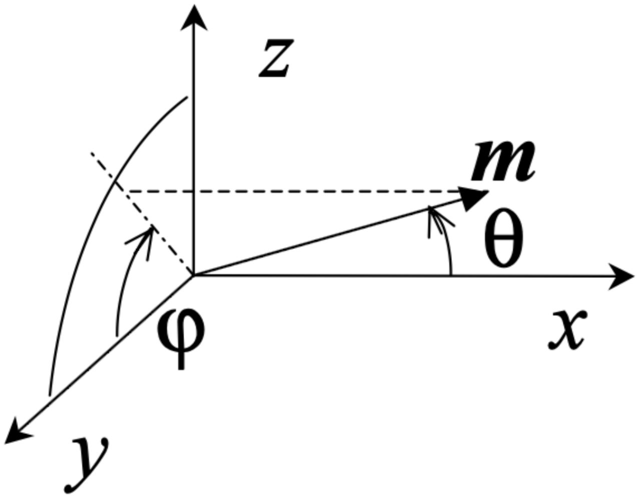

FIG. S5. Reference frame used to define the angles θ and φ of the magnetization in spherical coordinates.

an additional driving force that significantly increases the domain wall velocity after it exits the slit. Indeed, simulations confirm this mechanism, showing that the velocity upon exiting the slit can be up to twice its value prior to encountering the slit.

4 Extra Figures

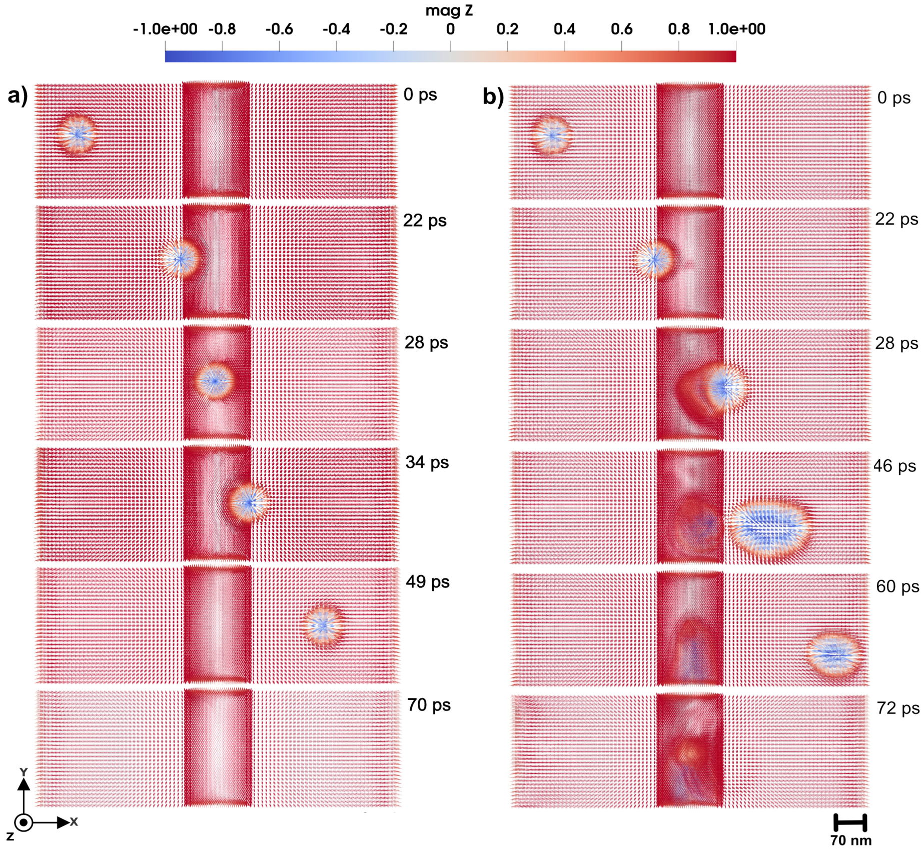

FIG. S6. 3D Skyrmion motion by applying STT of 15 m/s through the simulation cell, with a defect region shaped as a tetrahedron cluster with uniaxial anisotropy and a) hard axis along z-direction, b) easy axis along z-direction with strength of 0.11 mRy. The color bar is showing the z component of normalized magnetization. The dynamics of the system in a) is presented in Supplemental Material, Video 17 (2), and the dynamics of the system in b) is presented in Supplemental Material, Video 18 (2).

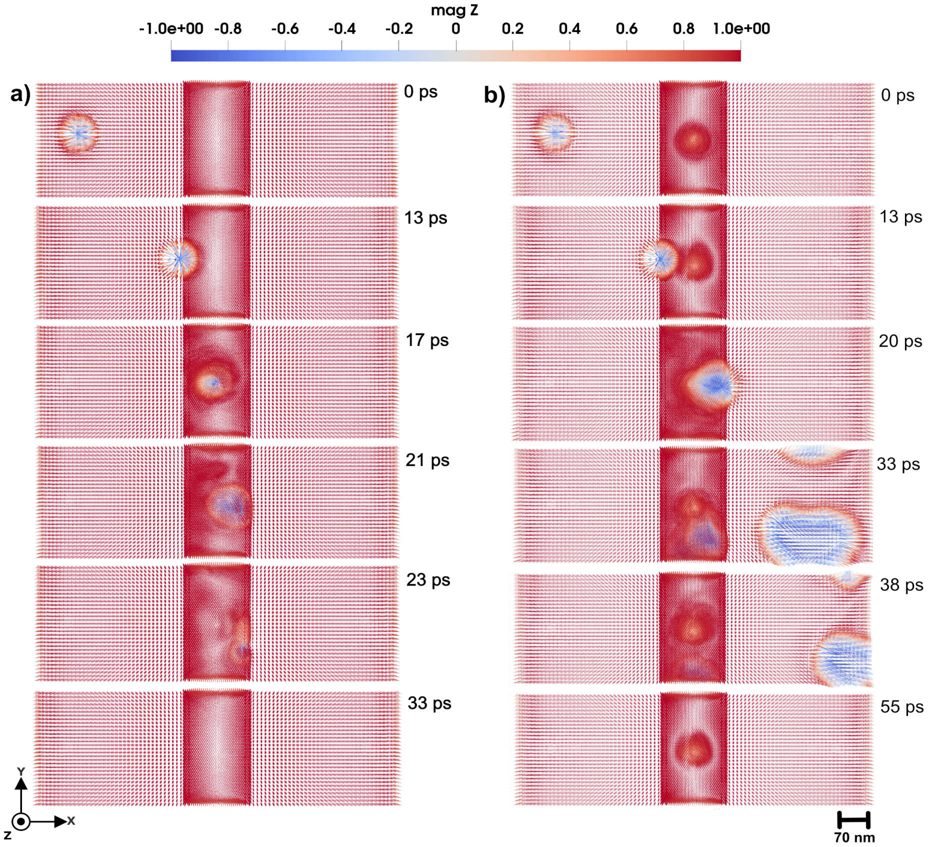

FIG. S7. 3D Skyrmion motion by applying STT of 25 m/s through the simulation cell, with a defect region shaped as a tetrahedron cluster with uniaxial anisotropy, a) hard axis along z-direction and b) easy axis along z-direction with strength of 0.9 mRy. The color bar is showing the z component of normalized magnetization. The dynamics of the system in a) is presented in Supplemental Material, Video 19 (2), and the dynamics of the system is presented in Supplemental Material, Video 20 (2).

References

1. Thiaville, A. & Nakatani, Y. Spin dynamics in confined magnetic structures iii. In Hillebrands, B. & Ounadjela, K. (eds.) *Spin Dynamics in Confined Magnetic Structures III*, 161–205 (Springer, Berlin, 2006).

2. See Supplementary Material at http://example.com/supplementary for detailed theory and technical aspects of the multiscale model. also the material parameters used in the simulations discussed in the main text are provided. Additionally, this resource includes videos illustrating the dynamics presented in Figs. 1, 2, 4–6, 8–10, and S6, S7. Videos included.
