# Supplementary material for: Hybrid micromagnetic and atomistic modeling of magnetization dynamics induced by engineered defects
Source: Sci Rep. 2025 Dec 21;15:44232. doi: 10.1038/s41598-025-31866-6 (PMC12722306; doi:10.1038/s41598-025-31866-6)
Supplement: Supplementary file 1 — Supplementary Information 1. [file 41598_2025_31866_MOESM1_ESM.zip › Revised-Supplementary/Supplementary Table Legend.pdf]

# Supplementary Table Legend

**Table S1:** Table of parameters used in the spin wave model to describe the diffraction pattern.
