# Supplementary material for: Hybrid micromagnetic and atomistic modeling of magnetization dynamics induced by engineered defects
Source: Sci Rep. 2025 Dec 21;15:44232. doi: 10.1038/s41598-025-31866-6 (PMC12722306; doi:10.1038/s41598-025-31866-6)
Supplement: Supplementary file 1 — Supplementary Information 1. [file 41598_2025_31866_MOESM1_ESM.zip › Revised-Supplementary/Supplementary Video Legends.pdf]

Supplementary Video Legends

Video S]1: Spin wave propagation through a 3D double-slit.
Video S2: 3D domain wall motion in the *x*-direction towards a double slit, induced by an applied external magnetic field.
Video S3: 3D Domain wall motion by applying magnetic field of 0.47 T in negative *z*-direction. In the center of the atomistic region a local cluster is placed that has uniaxial anisotropy with easy axis along the *z*-direction, with strength of 0.11 mRy.
Video S4: 3D Domain wall motion by applying magnetic field of 0.47 T in negative *z*-direction. In the center of the atomistic region a local cluster is placed that has uniaxial anisotropy with hard axis along the *z*-direction, with strength of 0.11 mRy.
Video S5: 3D Domain wall motion by applying magnetic field of 0.47 T in negative *z*-direction. In the center of the atomistic region a local cluster is placed that has uniaxial anisotropy with easy axis along *z*-direction and strength of 0.9 mRy.
Video S6: 3D Domain wall motion by applying magnetic field of 0.47 T in negative *z*-direction. In the center of the atomistic region a local cluster is placed that has uniaxial anisotropy with hard axis along *z*-direction and strength of 0.9 mRy.
Video S7: 3D Domain wall motion by applying a magnetic field of 1.5 T through a system with a tetrahedron cluster with uniaxial anisotropy that has easy axis along *z*-direction and strength of 0.9 mRy.
Video S8: 3D Domain wall motion by applying a magnetic field of 1.5 T through a system with a tetrahedron cluster with uniaxial anisotropy that has hard axis along *z*-direction and strength of 0.9 mRy.
Video S9: 3D Domain wall motion by applying a magnetic field of 2 T so that a domain wall moves through the tetrahedron cluster with uniaxial anisotropy that has easy axis along *z*-direction and strength of 1.5 mRy.
Video S10: 3D Domain wall motion by applying a magnetic field of 2 T so that a domain wall moves through the tetrahedron cluster with uniaxial anisotropy that has hard axis along *z*-direction and strength of 1.5 mRy.
Video S11: 3D Skyrmion motion by applying a STT of 30 m/s through the simulation cell, with a defect region provided by a tetrahedron cluster with uniaxial anisotropy. The easy axis is along the *z*-direction and the uniaxial anisotropy constant is 0.11 mRy.
Video S12: 3D Skyrmion motion by applying a STT of 30 m/s through the simulation cell, with a defect region provided by a tetrahedron cluster with uniaxial anisotropy. The hard axis is along the *z*-direction and the uniaxial anisotropy constant is 0.11 mRy.
Video S13: 3D Skyrmion motion by applying STT of 30 m/s through the simulation cell, with a defect region shaped as a tetrahedron cluster with uniaxial anisotropy. The easy axis is along the *z*-direction with the strength of 0.9 mRy.
Video S14: 3D Skyrmion motion by applying STT of 30 m/s through the simulation cell, with a defect region shaped as a tetrahedron cluster with uniaxial anisotropy. The hard axis is along the *z*-direction with the strength of 0.9 mRy.
Video S15: 3D Skyrmion motion by applying STT of 30 m/s through the simulation box, with a defect region shaped as a tetrahedron cluster with uniaxial anisotropy. The easy axis is along the *z*-direction with strength of 1.5 mRy.
Video S16: 3D Skyrmion motion by applying STT of 30 m/s through the simulation box, with a defect region shaped as a tetrahedron cluster with uniaxial anisotropy. The hard axis is along lying in *x*-*y* plane with strength of 1.5 mRy.
Video S17: 3D Skyrmion motion by applying STT of 15 m/s through the simulation cell, with a defect region shaped as a tetrahedron cluster with uniaxial anisotropy and hard axis along *z*-direction with strength of 0.11 mRy.
Video S18: 3D Skyrmion motion by applying STT of 15 m/s through the simulation cell, with a defect region shaped as a tetrahedron cluster with uniaxial anisotropy and easy axis along *z*-direction with strength of 0.11 mRy.
Video S19: 3D Skyrmion motion by applying STT of 25 m/s through the simulation cell, with a defect region shaped as a tetrahedron cluster with uniaxial anisotropy, the hard axis along *z*-direction with strength of 0.9 mRy.
Video S20: 3D Skyrmion motion by applying STT of 25 m/s through the simulation cell, with a defect region shaped as a tetrahedron cluster with uniaxial anisotropy, the easy axis along *z*-direction with strength of 0.9 mRy.
