# Supplementary figures and images for: Hybrid micromagnetic and atomistic modeling of magnetization dynamics induced by engineered defects
Source: Sci Rep. 2025 Dec 21;15:44232. doi: 10.1038/s41598-025-31866-6 (PMC12722306; doi:10.1038/s41598-025-31866-6)

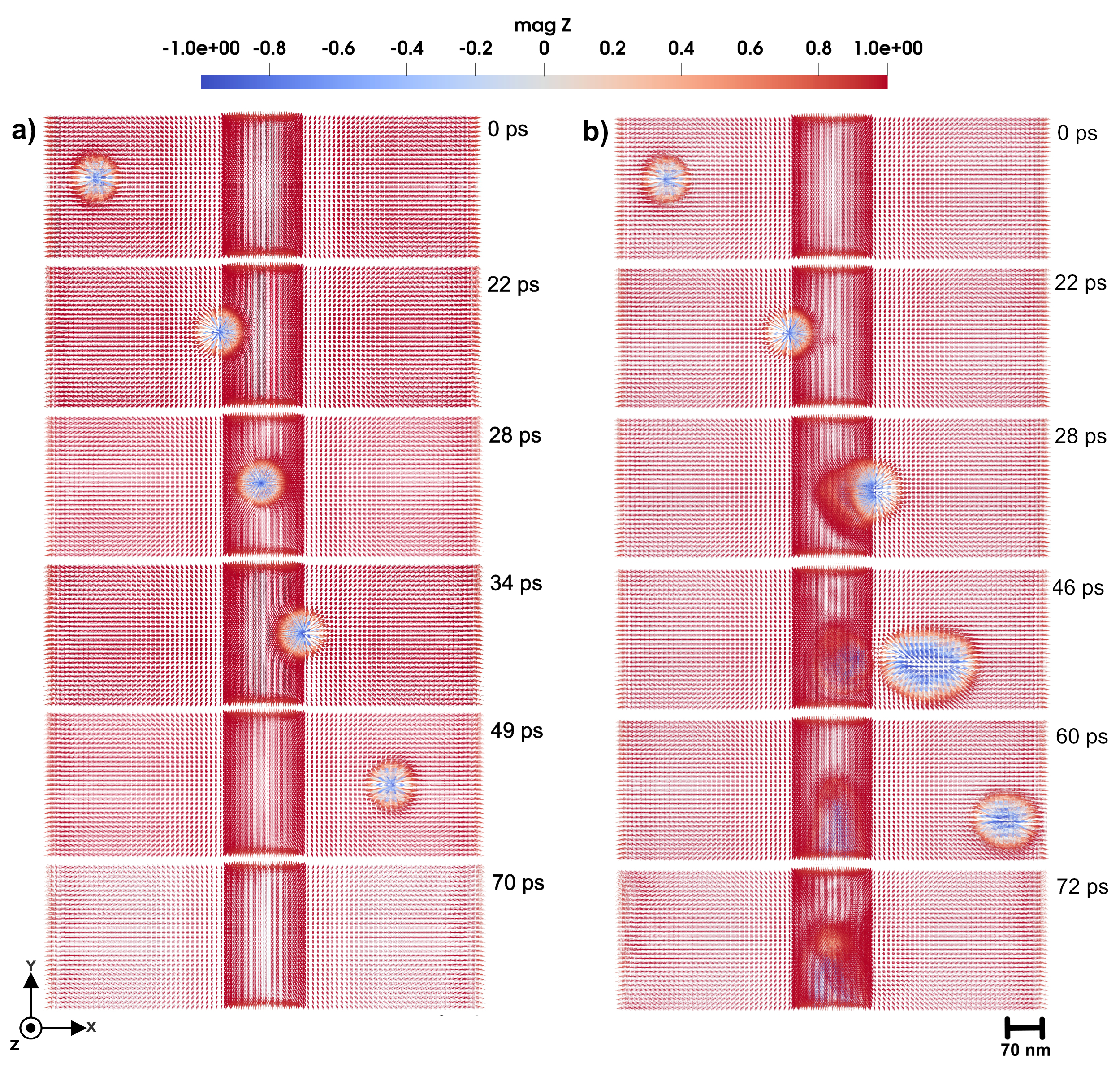

Supplement: Supplementary file 1 — Supplementary Information 1. [file 41598_2025_31866_MOESM1_ESM.zip › Revised-Supplementary/figS6.jpg]

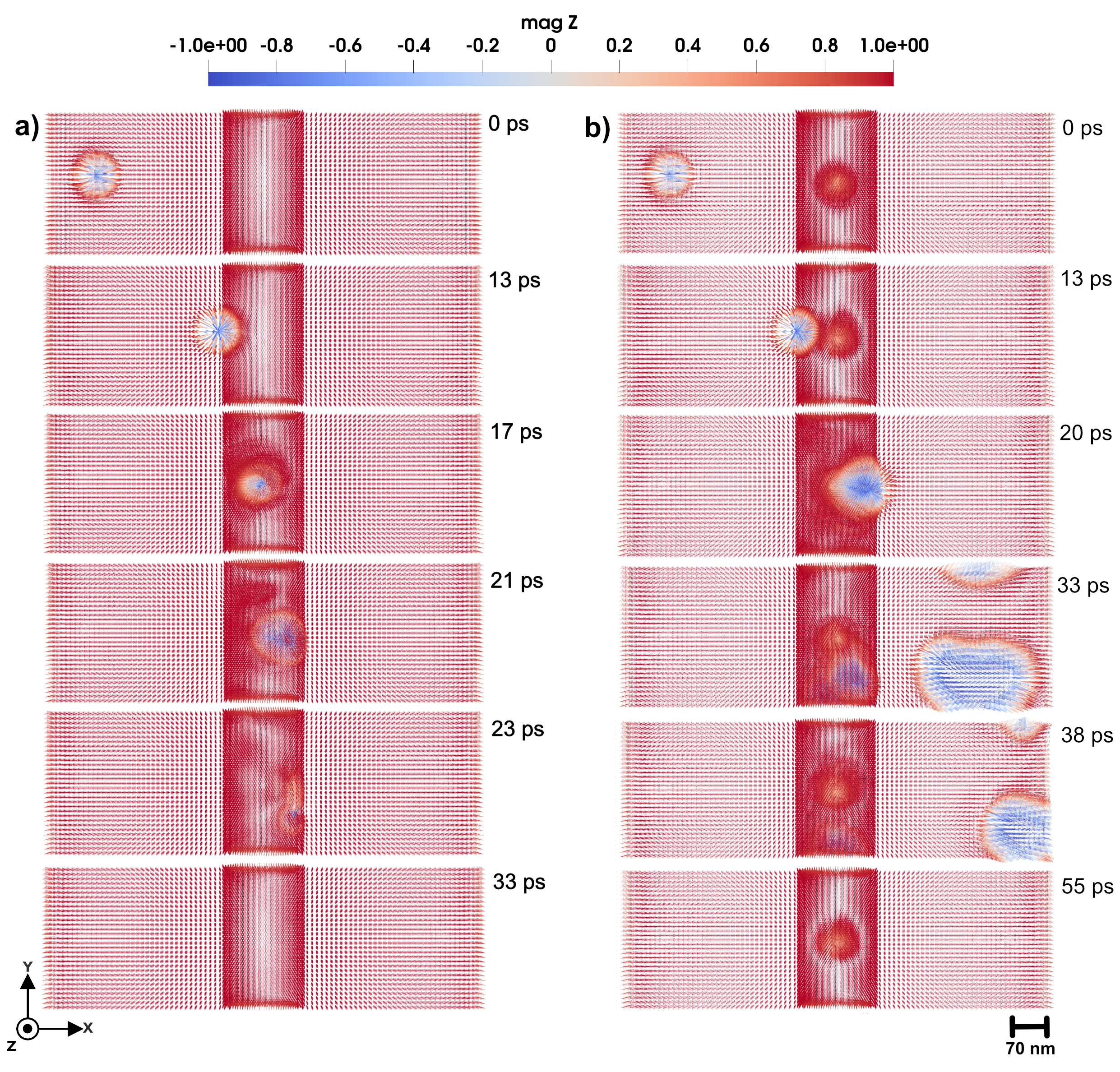

Supplement: Supplementary file 1 — Supplementary Information 1. [file 41598_2025_31866_MOESM1_ESM.zip › Revised-Supplementary/figS7.jpg]

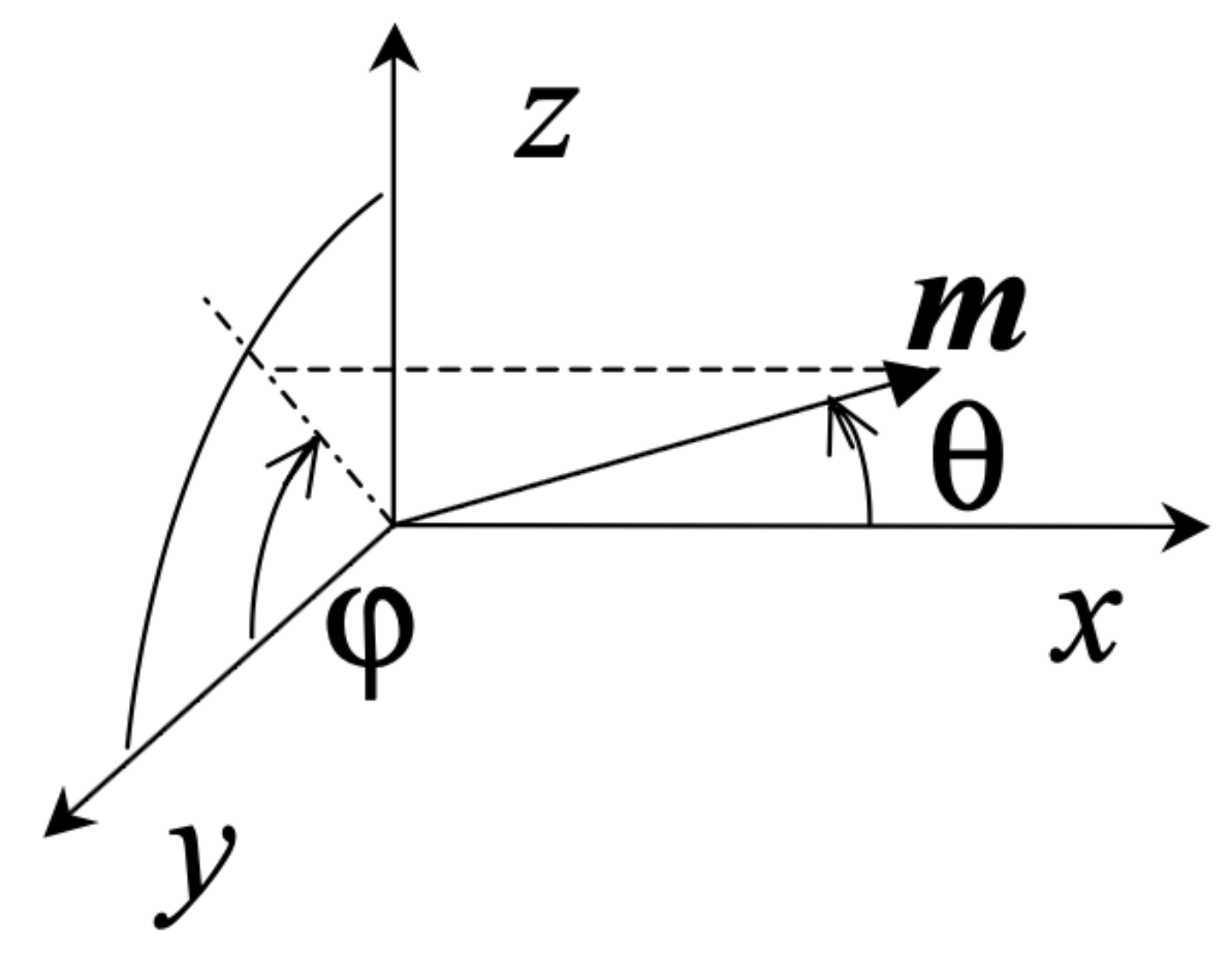

Supplement: Supplementary file 1 — Supplementary Information 1. [file 41598_2025_31866_MOESM1_ESM.zip › Revised-Supplementary/figS5.png]

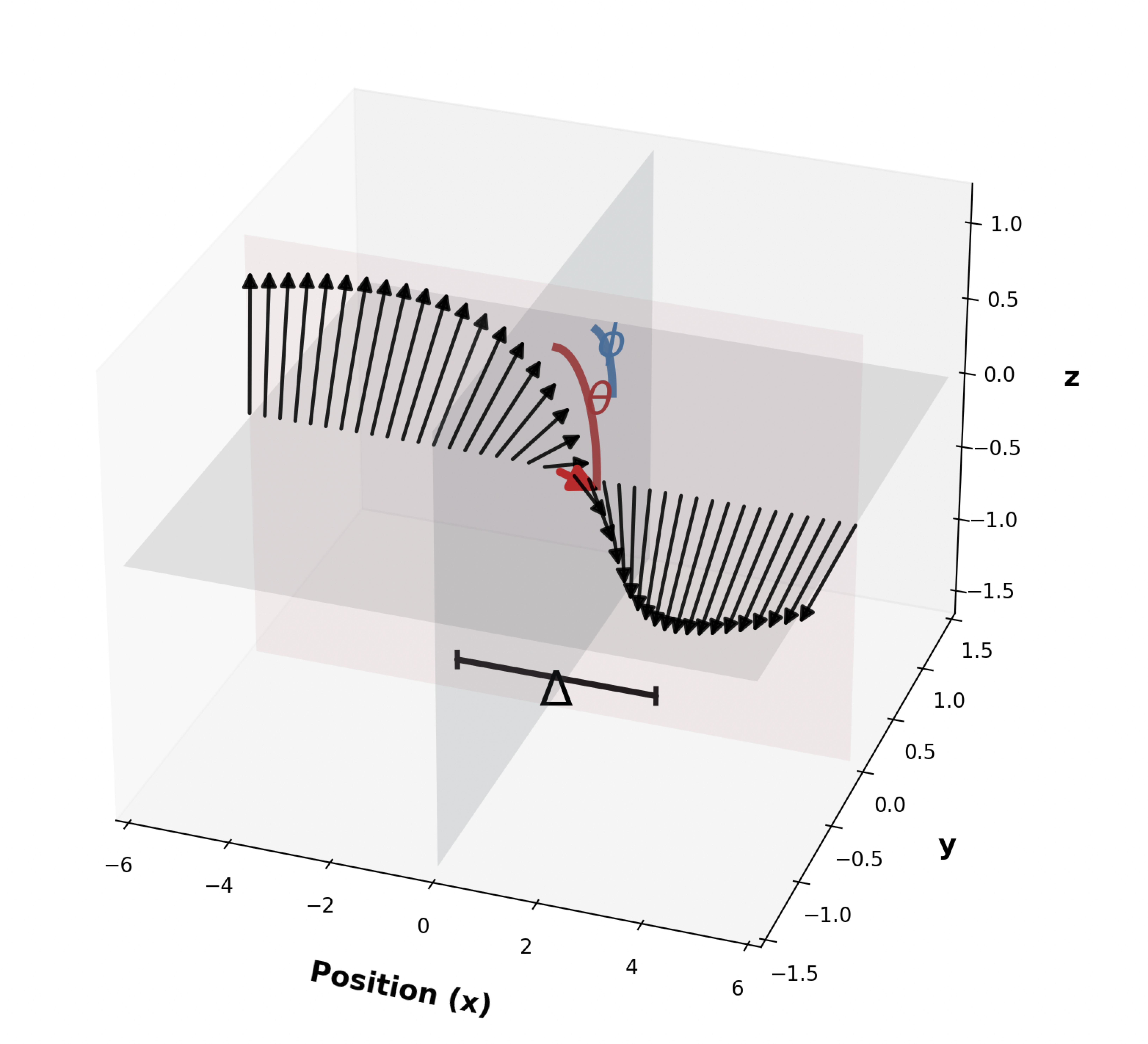

Supplement: Supplementary file 1 — Supplementary Information 1. [file 41598_2025_31866_MOESM1_ESM.zip › Revised-Supplementary/figS4.png]

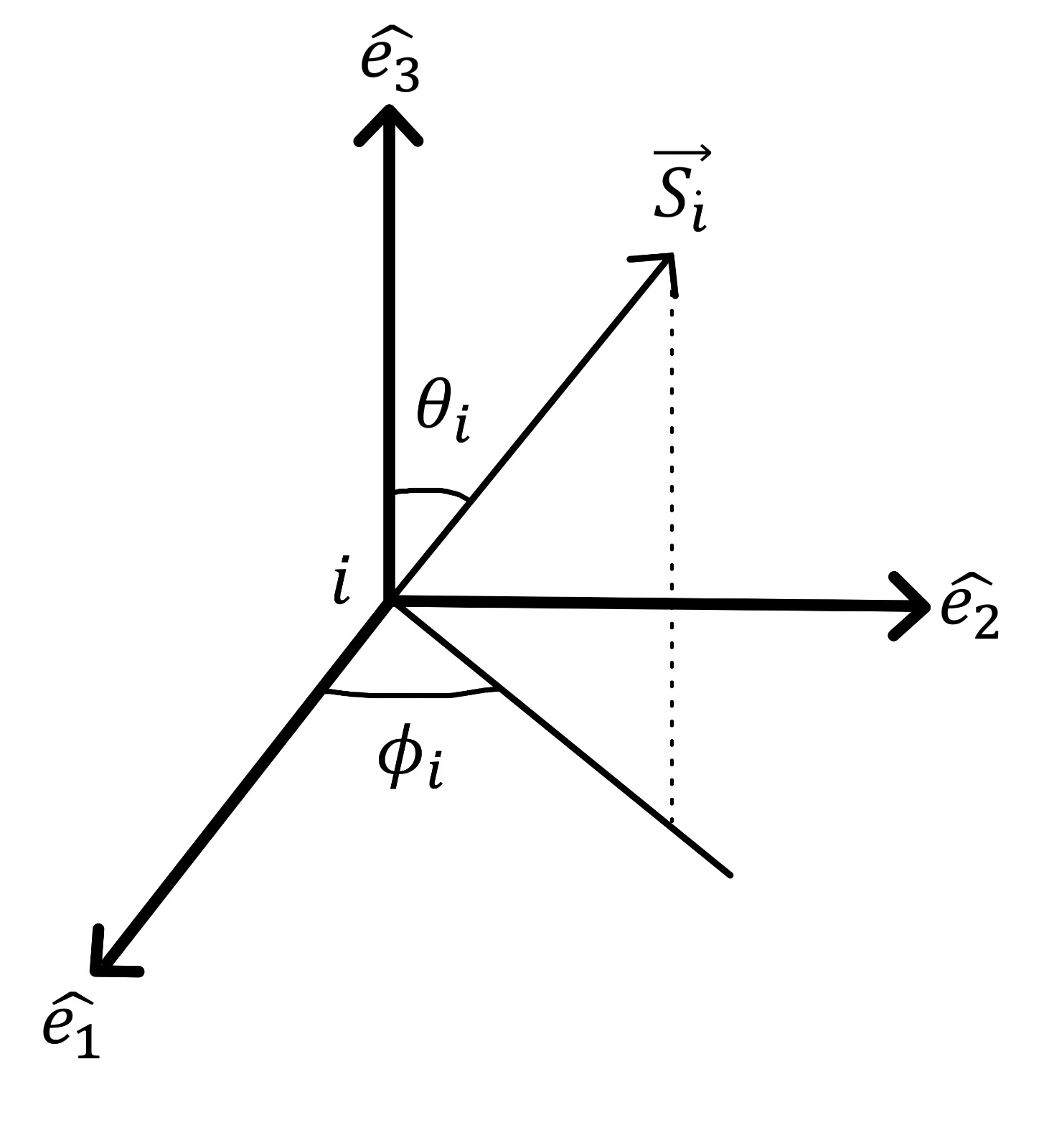

Supplement: Supplementary file 1 — Supplementary Information 1. [file 41598_2025_31866_MOESM1_ESM.zip › Revised-Supplementary/figS1.png]

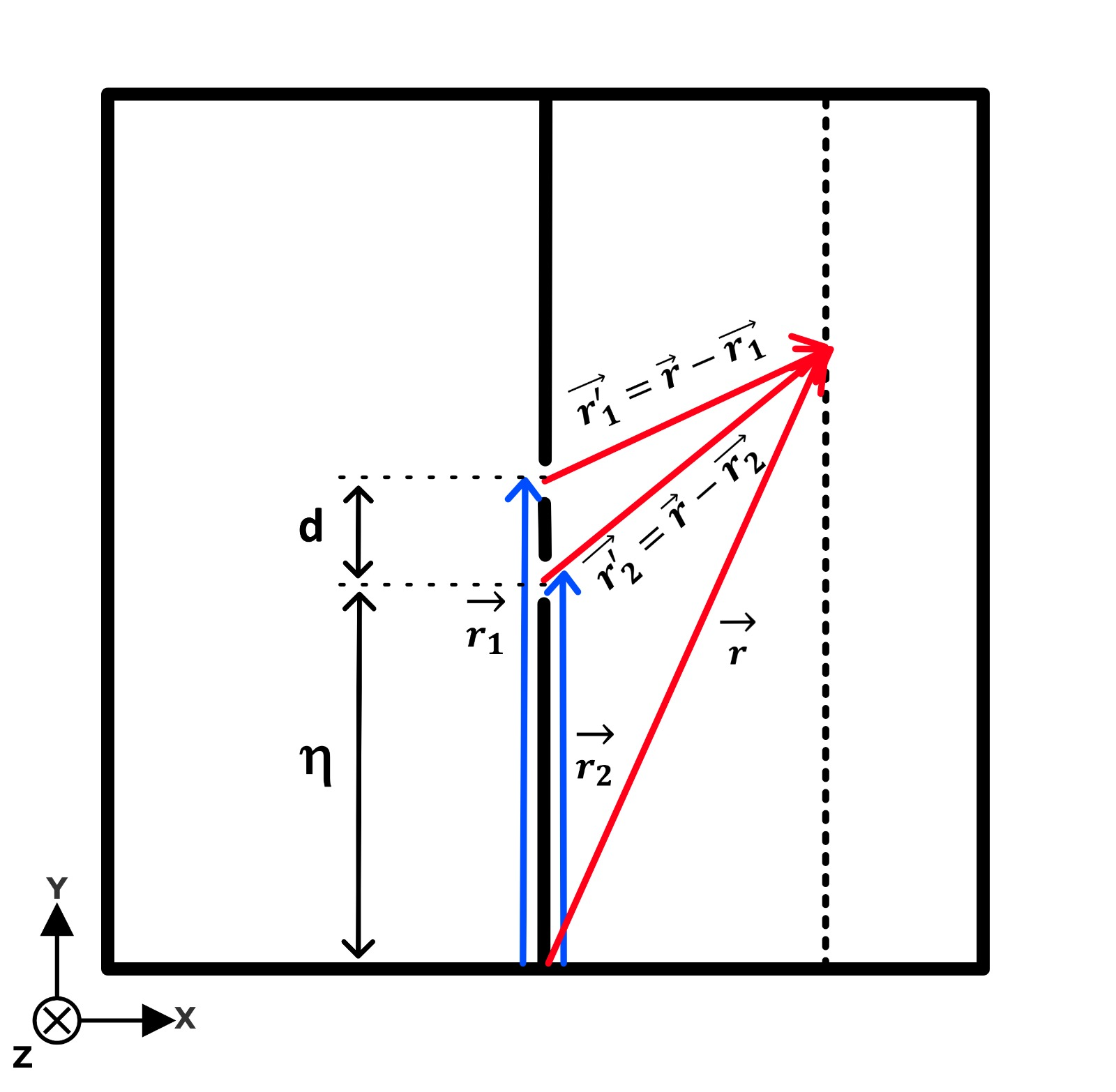

Supplement: Supplementary file 1 — Supplementary Information 1. [file 41598_2025_31866_MOESM1_ESM.zip › Revised-Supplementary/figS3.png]

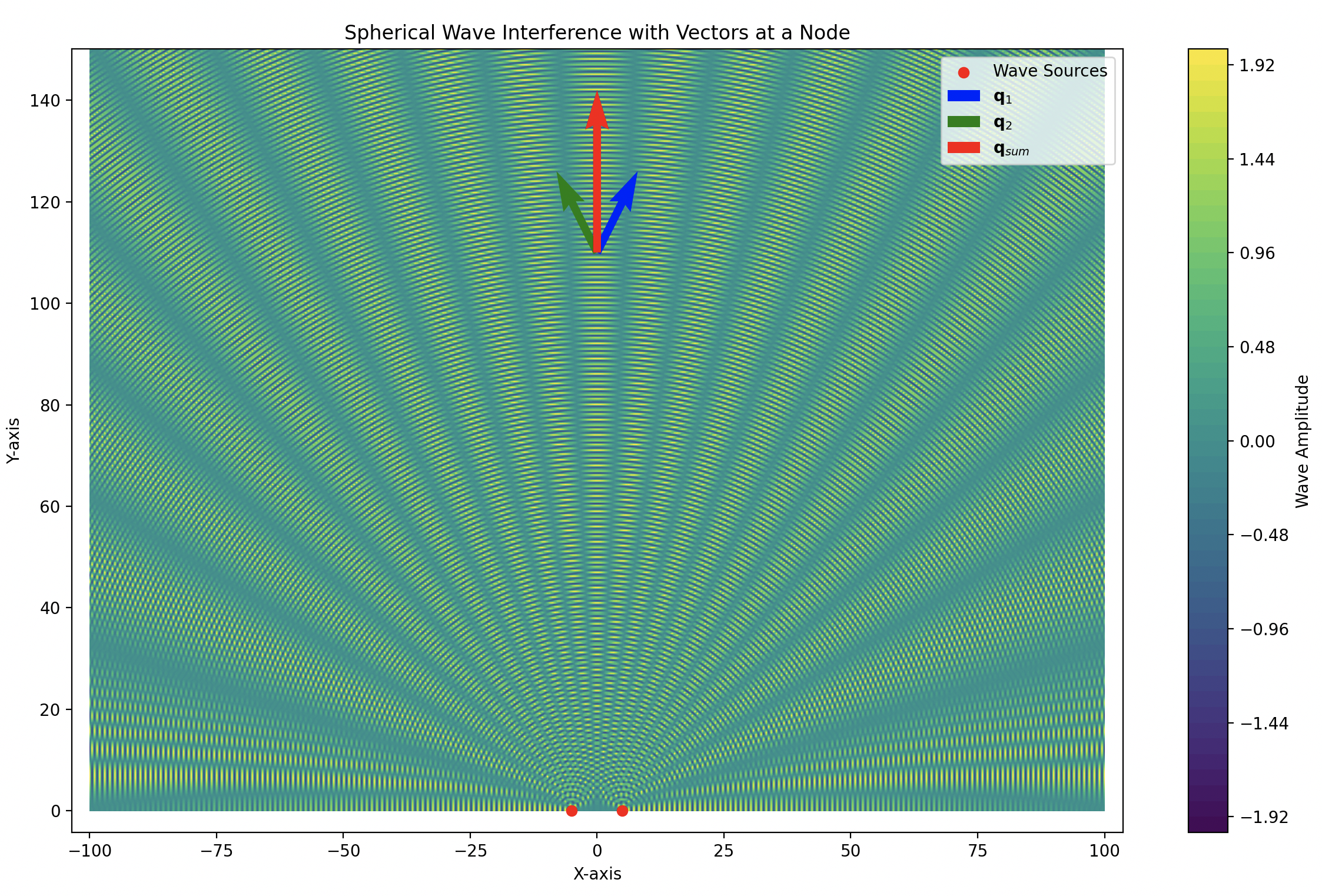

Supplement: Supplementary file 1 — Supplementary Information 1. [file 41598_2025_31866_MOESM1_ESM.zip › Revised-Supplementary/figS2.png]
